# Multi-omics analysis on the mechanism of the effect of Isatis leaf on the growth performance of fattening sheep

**DOI:** 10.3389/fvets.2024.1332457

**Published:** 2024-02-07

**Authors:** Zhikun Cao, Mingliang Yi, Jialu Zhou, Zhiyu Zhang, Zibo Liu, Chao Yang, Shixin Sun, Lei Wang, Yinghui Ling, Zijun Zhang, Hongguo Cao

**Affiliations:** ^1^College of Animal Science and Technology, Anhui Agricultural University, Hefei, China; ^2^Anhui Province Key Laboratory of Local Livestock and Poultry Genetic Resource Conservation and Bio-Breeding, Anhui Agricultural University, Hefei, China

**Keywords:** Isatis leaf (ISL), fattening sheep, growth performance, multi-omics, immunity

## Abstract

**Introduction:**

This study evaluated the effects of Isatis Leaf (ISL) on the growth performance, gastrointestinal tissue morphology, rumen and intestinal microbiota, rumen, serum and urine metabolites, and rumen epithelial tissue transcriptome of fattening sheep.

**Methods:**

Twelve 3.5-month-old healthy fattening sheep were randomly divided into two groups, each with 6 replicates, and fed with basal diet (CON) and basal diet supplemented with 80 g/kg ISL for 2.5 months. Gastrointestinal tract was collected for histological analysis, rumen fluid and feces were subjected to metagenomic analysis, rumen fluid, serum, and urine for metabolomics analysis, and rumen epithelial tissue for transcriptomics analysis.

**Results:**

The results showed that in the ISL group, the average daily gain and average daily feed intake of fattening sheep were significantly lower than those of the CON group (*P* < 0.05), and the rumen ammonia nitrogen level was significantly higher than that of the CON group (*P* < 0.01). The thickness of the reticulum and abomasum muscle layer was significantly increased (*P* < 0.05). At the genus level, the addition of ISL modified the composition of rumen and fecal microorganisms, and the relative abundance of Methanobrevibacter and Centipeda was significantly upregulated in rumen microorganisms, The relative abundance of Butyrivibrio, Saccharofermentans, Mogibacterium, and Pirellula was significantly downregulated (*P* < 0.05). In fecal microorganisms, the relative abundance of Papillibacter, Pseudoflavonifractor, Butyricicoccus, Anaerovorax, and Methanocorpusculum was significantly upregulated, while the relative abundance of Roseburia, Coprococcus, Clostridium XVIII, Butyrivibrio, Parasutterella, Macellibacteroides, and Porphyromonas was significantly downregulated (*P* < 0.05). There were 164, 107, and 77 different metabolites in the rumen, serum, and urine between the ISL and CON groups (*P* < 0.05). The differential metabolic pathways mainly included thiamine metabolism, niacin and nicotinamide metabolism, vitamin B6 metabolism, taurine and taurine metabolism, beta-Alanine metabolism and riboflavin metabolism. These metabolic pathways were mainly involved in the regulation of energy metabolism and immune function in fattening sheep. Transcriptome sequencing showed that differentially expressed genes were mainly enriched in cellular physiological processes, development, and immune regulation.

**Conclusion:**

In summary, the addition of ISL to the diet had the effect of increasing rumen ammonia nitrogen levels, regulating gastrointestinal microbiota, promoting body fat metabolism, and enhancing immunity in fattening sheep.

## 1 Introduction

The animal husbandry industry has entered a new era of healthy farming, and in order to promote the development of healthy livestock and poultry farming, finding natural feed additives to replace antibiotics has become a research hotspot. Plant additives contain bioactive substances such as alkaloids, saponins, volatile oils, tannins, and polysaccharides, which have various functions such as sterilization, growth promotion, and oxidation resistance. They are considered one of the natural feed additives as substitutes for feed antibiotics (1–4). In recent years, research on the nutritional regulation and production application of Chinese herbal medicine in livestock and poultry has received widespread attention (5, 6). Research has found that Chinese herbal feed additives can improve the growth performance and carcass quality of livestock and poultry (7–9). Different Chinese herbal medicines have a certain promoting effect on the growth performance of livestock, and the main effect on livestock is to improve the structure of beneficial bacteria in the gastrointestinal tract, enhance nutrient absorption levels and immune performance in the gastrointestinal tract (10–12). As one of the most common herbaceous plants, ISL is widely distributed around the world. As a high-yield, efficient, high-quality, and high-economic crop, it has rich nutritional value and is widely used in industries such as food, traditional Chinese medicine, dietary therapy, and health products (13).

The main active substances contained in ISL include three types of compounds: indoles (indigo, indirubin), quinazolones (tryptamines), and glucosinolates. These active substances have antibacterial, anti-inflammatory, antiviral, and immune regulating effects (14–16). In addition, ISL is rich in cellulose, which helps gastrointestinal peristalsis, promotes food digestion, increases satiety, and lowers cholesterol, reduces the accumulation of fat in the body. Research has found that the extract of ISL has multiple effects on inhibiting skin fibroblast aging by regulating mTOR-NF-κB-SASP signaling (17), reduces stress-induced behavior and cellular disorders in mice through antioxidant and anti-inflammatory effects (18), and the extract also has anti-wrinkle effects (19). ISL is a common herbaceous plant and Chinese herbal medicine, but there is currently no in-depth study on the growth performance of fattening sheep fed with ISL. This experiment studied the effect of 8% ISL diet on the growth performance of fattening sheep, and further explored the mechanism of ISL on the dynamic changes of rumen and intestinal microbiota, serum antioxidant capacity, metabolome (rumen fluid, serum and urine), and transcriptome in fattening sheep.

## 2 Material and methods

### 2.1 Experimental animals and experimental design

**Figure 1:**
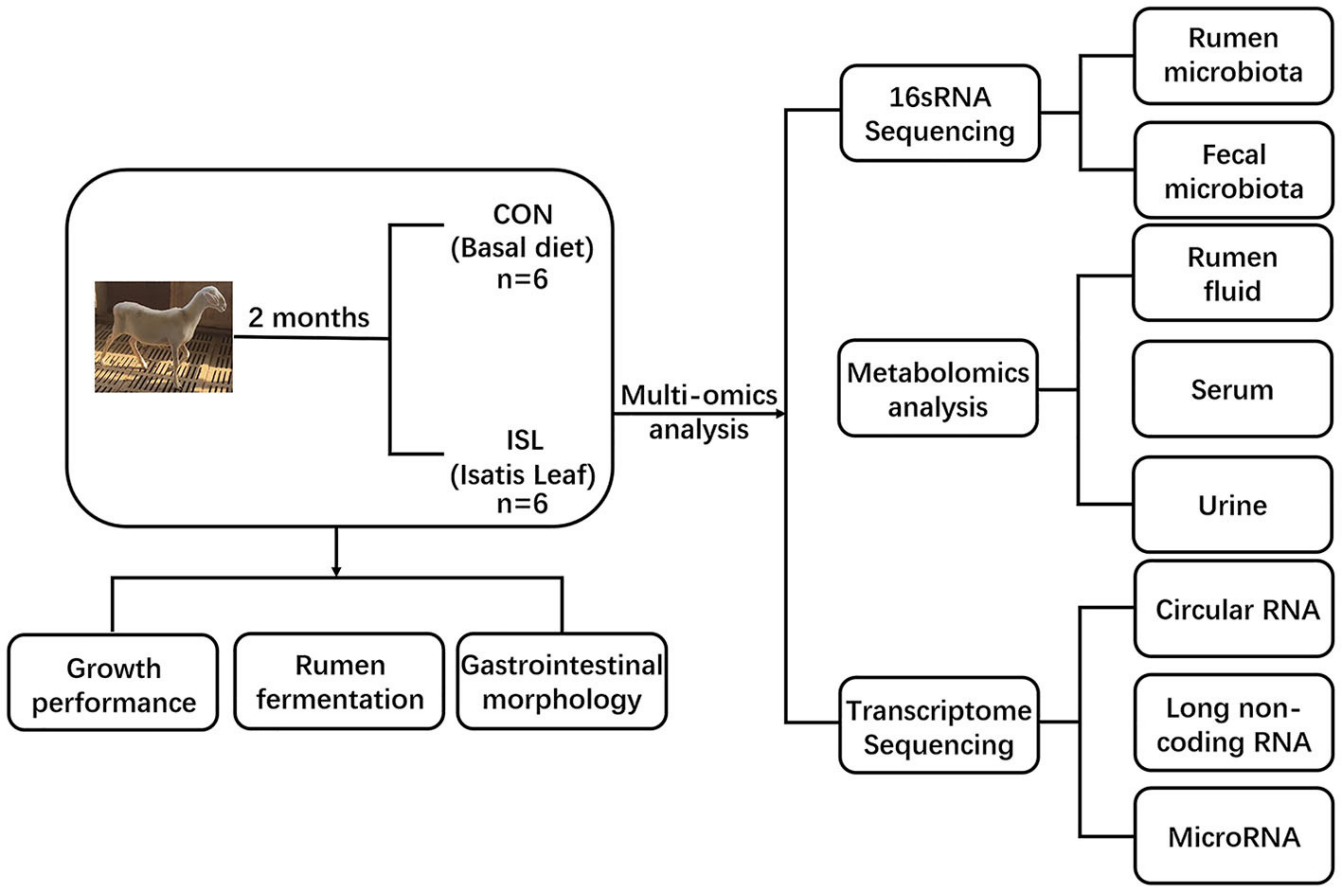
Experimental design and workflow of feeding fattening sheep with Isatis leaf diet. Including rumen and fecal microbiome, rumen fluid, serum and urine metabolome, and transcriptome sequencing of rumen epithelial cells. Twelve Hu sheep were randomly assigned to a basal diet (CON) or a basal diet supplemented with 80 g/kg Isatis leaf DM (ISL).

The experiment selected 12 healthy 3.5-month-old Hu sheep (Chinese native sheep breeds) with an average weight of 22.42 ± 2.15 kg and randomly divided into two groups, with 6 sheep in each group. The feeding experiment was conducted at Tianchang Zhoushi Sheep Industry Co., Ltd. (Chuzhou, China). The CON was fed a basic diet, while the ISL was fed a basic diet replaced by 8% ISL. The ingredient composition is shown in Table 1. The experimental period was 2.5 months, and the pre-feeding period was 0.5 months. Before the experiment, the enclosure was disinfected and ventilated, routine epidemic prevention measures such as insect repellent were carried out on the experimental sheep, and fattening sheep were fed at fixed times in the morning and evening every day, with free feeding and drinking water. The experimental plan was approved by the Animal Protection Committee of Anhui Agricultural University (NO: SYDW–P20190600601), and the experimental design and workflow are shown in the Figure 1.

**Table 1:** Main components of the diet for fattening sheep during the experimental period.

**Item**	**CON**	**ISL**
**Ingredient (%)**		
Ground corn grain	28.00	25.76
Soybean meal	15.00	13.80
Rapeseed meal	9.00	8.28
Wheat bran	4.00	3.68
Sodium bicarbonate	1.00	0.92
Salt	1.00	0.92
Dicalcium phosphate	0.50	0.46
Calcium carbonate	0.50	0.46
Premix^a^	1.00	0.92
Isatis tinctoria L. leaf	0.00	8.00
Peanut straw	15.00	13.80
Soybean straw	25.00	23.00
**Chemical composition (%, DM)**		
Organic matter	91.30	91.56
CP	15.10	15.28
NDF	38.70	39.88
ADF	23.20	25.61
Ether extract	3.10	2.91
Calcium	0.75	0.75
Phosphorus	0.43	0.43
Metabolizable energy^b^, MJ/Kg	9.83	9.77

CON, control diet; ISL, isatis leaf.

^a^Formulated to provide (per kilogram of premix) 600 KIU of Vitamin A, 80 KIU of vitamin D3, 5,000 IU of Vitamin E, 8,000 mg of Zn, 60 mg of Se, 200 mg of I, 9,400 mg of Fe, 72 mg of Co, 10,400 mg of Mn, and 1,600 mg of Cu.

^b^Calculated according to Ministry of Agriculture of P.R. China, 2004.

### 2.2 Experimental samples and data collection

Three feed samples were collected per week (Monday, Wednesday, and Friday), and each sheep feed sample was mixed and sampled twice. The samples were dried at 85°C for 2,880 min and filtered through a 1 mm sieve to analyze dry matter (DM), crude protein (CP) and ash (20), as well as neutral derivative fiber (NDF) and acid derivative fiber (ADF) (21). On the first day of the formal experiment of fattening sheep, the body weight before morning feeding was the initial body weight. On the last day of the formal experiment, the test sheep were weighed before morning feeding, and the average daily gain of each stage was calculated. The daily feeding amount and leftover amount were accurately recorded, and the average daily feed intake was calculated. The feed conversion rate was calculated based on the average daily gain/average daily feed intake.

On the last morning of the feeding experiment, samples of rumen fluid, feces, serum, and urine were collected from fattening sheep. A total of 12 fattening sheep in the ISL and CON groups were collected with a rumen fluid sampler. The collected rumen fluid was filtered with 4 layers of gauze, and 1 mL of each sample was analyzed for volatile fatty acids (VFAs) (22). Two mL was used for colorimetric analysis of ammonia nitrogen content (23). The remaining liquid nitrogen was immediately stored in a liquid nitrogen tank for future use after being transferred to a frozen storage tube. Blood was collected from the jugular vein of the blood collection vessel on an empty stomach and allowed to stand at room temperature for 120 min. The serum was centrifuged at 3500 r/min for 15 min. After being drawn, it was divided into 1.5 mL centrifuge tubes and stored at −20°C. Fresh urine from 12 fattening sheep was collected and divided into 2 mL cryotubes, which were stored in liquid nitrogen. The fresh feces of the fattening sheep were collected using the rectal fecal collection method. The feces were immediately placed in a 15 mL centrifuge tube and stored in liquid nitrogen for storage.

After the feeding experiment, the fasted fattening sheep were slaughtered, and the rumen epithelial tissue was collected. After being washed with physiological saline, it was placed in an EP tube and quickly stored in liquid nitrogen for transcriptome sequencing. The collected gastrointestinal tissue was fixed with 4% paraformaldehyde, and histological sections were made using HE staining following the steps of fixation, dehydration, transparency, wax immersion, embedding, sectioning, staining, and sealing. After neutral gum sealing, the tissue sections were observed under a microscope, and disposable sterile gloves and masks were worn during sample collection to avoid contamination.

### 2.3 16S rRNA sequencing of rumen and fecal microorganisms

The rumen fluid and fecal samples were thawed at 4°C, and the total DNA was extracted with E.Z.N.A. fecal DNA kit (D4015, Omega Bio tek, Norcross, GA, United States). The concentration and purity of DNA samples were determined under the condition of OD260/OD280 absorbance, and DNA integrity was detected by 1% agarose gel electrophoresis. PCR amplification was performed on the V3-V4 variable region of the bacterial 16S rRNA gene, with primer sequences 515F (5′- CCTACACGACGTCTTCCGATCTN-3′) and 806R (5′- GACTGGATCCCTTGACCCGATTCCA-3′). A total of 30 μL PCR amplification reaction system: 15 μL 2 × Phanta Master Mix; 1 μL Bar PCR prime F (10 μM), 1 μL prime R (10 μM), 10 ng Genomic DNA, supplemented with ddH_2_O to 30 μL. Amplification conditions: pre-denaturation at 95°C for 5 min; 95°C denaturation for 0.5 min, 55°C annealing for 0.5 min, 72°C extension for 0.75 min, 27 cycles; After extending at 72°C for 10 min, the PCR products were collected for detection and purification, and sent to Sangon (Shanghai, China) for sequencing based on the Illumina Miseq platform. After quality control and processing of the original sequencing data, the differences in microbial diversity and community structure in the rumen and feces of fattening sheep in the CON and ISL groups were analyzed.

### 2.4 Metabolomics detection of rumen fluid, serum, and urine

Frozen rumen fluid, serum, and urine samples were sent to Shanghai Baiqu Biotechnology Co., Ltd. for metabolomics testing and analysis, including sample processing, non-targeted serum metabolomics testing, and data processing analysis. The rumen fluid, serum, and urine samples were thawed, and 50 μL of the sample was mixed with 150 μL of pre-cooled ice methanol (containing 1 μg/mL of 2-Chlorophenylalanine). The supernatant was centrifuged at 4°C at 12,000 r/min for 10 min, and metabolomics analysis was performed using ultra-high-performance liquid chromatography-tandem quadrupole time-of-flight mass spectrometry (UPLC-Q-TOF-MS). Chromatographic column: ACQUITY UPLC HSS T3 column (100 mm × 2.1 mm, 1.8 μm); The mobile phase A is ultrapure water (0.1% formic acid), and the B phase is acetonitrile (0.1% formic acid); Elution gradient: 0–1 min (2% B), 1–2 min (2%–5% B), 2–5 min (5%–12% B), 5–10 min (12%–20% B), 10–12 min (20%–30% B), 12–13 min (30%–50% B), 13–15 min (50%–100% B). Flow rate of 0.5 mL/min, column temperature of 40°C, injection volume of 5 μL. The ionization source is an electric spray ionization source (ESI) in positive and negative ion scanning mode. The raw data obtained from UPLC-Q-TOF-MS sequencing was processed and differential metabolites were screened under the conditions that the *p*-value of Student's *t*-test was < 0.05, and the Variable Importance in the Projection (VIP) of the first principal component in the OPLS-DA model was >1. Metabolic pathway enrichment analysis was performed on the screened differential metabolites.

### 2.5 Transcriptomics analysis

The rumen epithelial tissue samples preserved in liquid nitrogen were sent to Shanghai Shenggong Biotechnology Co., Ltd. in China for transcriptome sequencing. The total RNA of rumen epithelial tissue was extracted with Trizol reagent, the concentration and purity of total RNA (OD 260 nm/OD 280 nm and OD 260 nm/OD 230 nm) were detected with Nanodrop 2000 ultra micro spectrophotometer, the RNA integrity was detected with 1% agarose gel electrophoresis, and the qualified RNA was stored in an ultralow temperature refrigerator at −80°C for standby.

High throughput sequencing was performed using the Illumina Hiseq™ sequencing platform, and the raw data obtained from sequencing was evaluated and controlled for quality. FastQC was used for quality evaluation, Trimmatic was used for quality pruning, and DESeq2 was used for significance analysis of samples. The screening conditions were set to *P* < 0.05 and |fold change|>2 and the differentially expressed genes (DEGs) obtained were subjected to Gene ontology (GO) enrichment analysis using ClusterProfiler software.

### 2.6 Statistical analysis

We used Excel to organize the data, and SPSSAU data analysis platform (https://spssau.com/) was used for data normal distribution testing. SPSS software independent sample *T*-test was used for statistical analysis, with *P* < 0.05 indicating significant differences and *P* < 0.01 indicating extremely significant differences. The test sample consists of 6 replicates.

## 3 Results

### 3.1 Growth performance

5Before the formal experiment, we measured the weight of fattening sheep in the CON and ISL groups. The initial average weight of the ISL group was 23.31 ± 1.98 kg, while the CON group was 23.25 ± 1.54 kg, with no significant difference. After 2 months of feeding, the average daily gain of fattening sheep in the ISL group and CON group was 0.18 ± 0.02 kg and 0.21 ± 0.01 kg. The average daily gain (ADG) of fattening sheep in the ISL group was significantly lower than that in the CON group (*P* < 0.05). The average daily feed intake (ADFI) of the ISL group was 1.03 ± 0.05 kg, while the CON group was 1.21 ± 0.04 kg. The average daily feed intake was significantly reduced (*P* < 0.05), and the feed conversion rate was both 0.17 ± 0.01, with no significant difference. During the fattening period when 8% ISL was added to the diet, the fattening sheep's body was healthy. Further research is needed to investigate the mechanism of the influence of ISL on the growth of fattening sheep.

### 3.2 Gastrointestinal morphology

**Figure 2:**
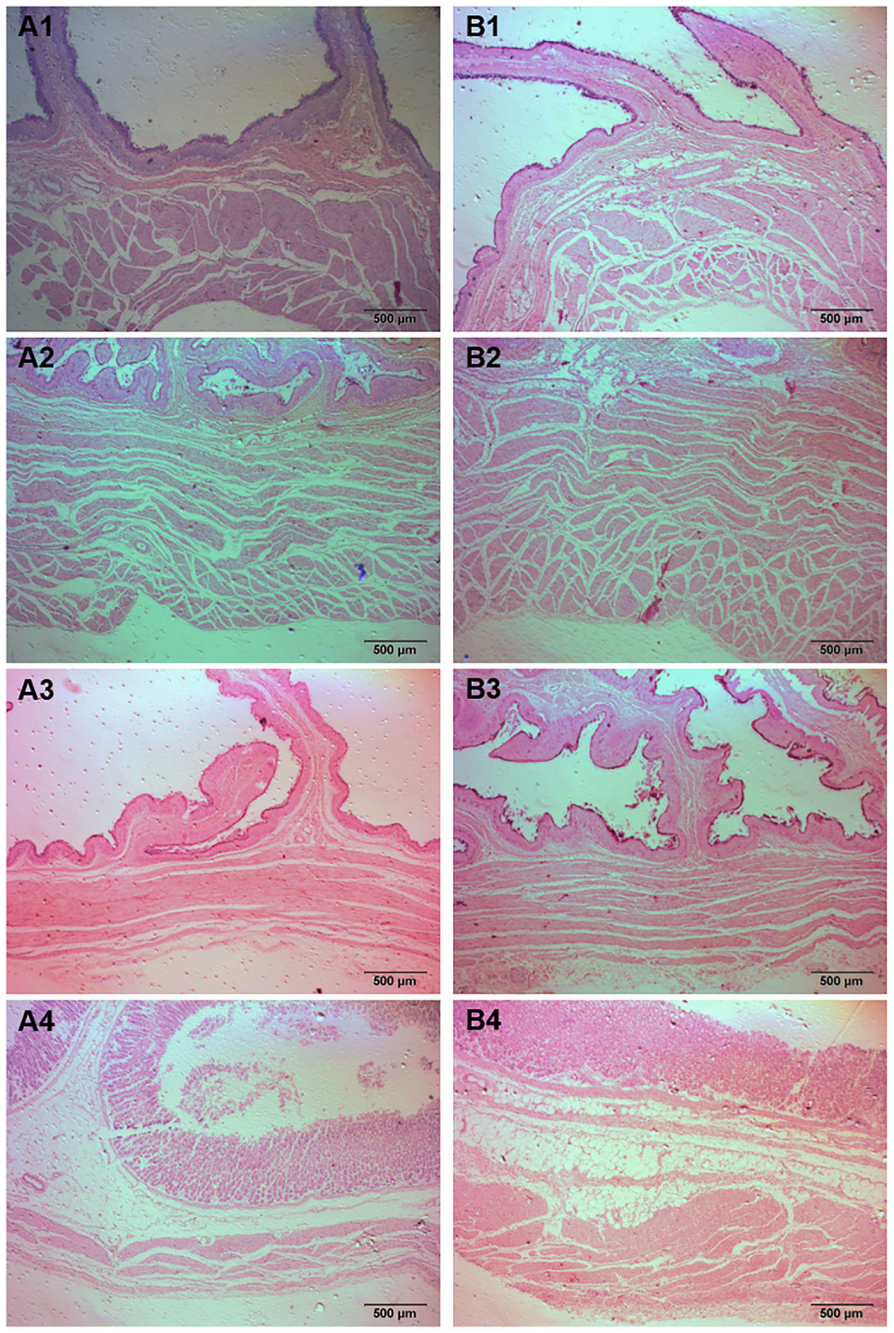
Histomorphology of stomachs in fattening sheep fed with ISL diet. **(A)** CON, **(B)** ISL, (1) Rumen, (2) Reticulum, (3) Omasum, (4) Abomasum.

The gastrointestinal tract of animals is the place where nutrients are digested and absorbed, and changes in its organizational structure are extremely important for animal feed intake and digestion and absorption capacity (24). At present, there are limited reports on the effects of ISL on the gastrointestinal development of fattening sheep. We selected the gastrointestinal tissue of the ISL group and the CON group fattening sheep to observe the morphological structure, and measured their muscle layer thickness (Figure 2, Supplementary Figure S1). The measurement results and statistical data are shown in the Table 2 and Supplementary Table S1. Compared with the CON group, the thickness of the reticulum and abomasum muscle layers in the ISL group was significantly increased (*P* < 0.05), and there was no significant change in the thickness of the rumen and omasum muscle layers. The difference in muscle layer thickness and villus length of each intestinal segment tissue was not significant.

**Table 2:** Effect of ISL on the morphology of gastric tissue in fattening sheep.

**Position**	**Item**	**CON (um)**	**ISL (um)**
Rumen	Muscularis	1,010.62 ± 34.26	979.56 ± 27.46
Reticulum	Muscularis	1,335.28 ± 76.93^b^	1,926.77 ± 136.66^a^
Omasum	Muscularis	706.78 ± 10.17	728.23 ± 55.15
Abomasum	Muscularis	451.64 ± 21.53^b^	746.15 ± 42.11^a^

^a,b^Values within a row with different superscripts differ significantly at *P* < 0.05.

### 3.3 Rumen fermentation and microbiota

Ammonia nitrogen is the main nitrogen source for rumen microbial fermentation and an important indicator affecting microbial activity. Suitable ammonia nitrogen is the primary condition to ensure the efficiency of microbial protein synthesis (25, 26). The concentration of ammonia nitrogen and volatile fatty acid (VFA) in the rumen are important indicators reflecting the fermentation status of feed in the rumen. After adding ISL feed, it was found that the levels of ammonia nitrogen in the ISL group were significantly higher than those in the CON group (*P* < 0.01). The total VFA, acetic acid, propionic acid, butyric acid, valeric acid, and acetic acid and propionic acid concentrations in the ISL group were increased compared to the CON group, but the difference was not significant (Table 3).

**Table 3:** Rumen fermentation characteristics of Isatis leaf diet treatment.

**Item**	**Treatment**		**SEM**	***P*-value**
	**CON**	**ISL**		
Ammonia-nitrogen, mg/dL	6.21^b^	14.01^a^	1.865	0.001
Total volatile fatty acid, mM	66.10	68.94	2.709	0.311
Acetate, mM	45.23	47.57	2.555	0.374
Propionate, mM	12.21	12.54	0.844	0.704
Butyrate, mM	7.85	8.02	0.497	0.734
Valerate, mM	0.81	0.81	0.050	0.976
Acetate: propionate	3.71	3.94	0.386	0.565

^a,b^Values within a row with different superscripts differ significantly at *P* < 0.05.

**Figure 3:**
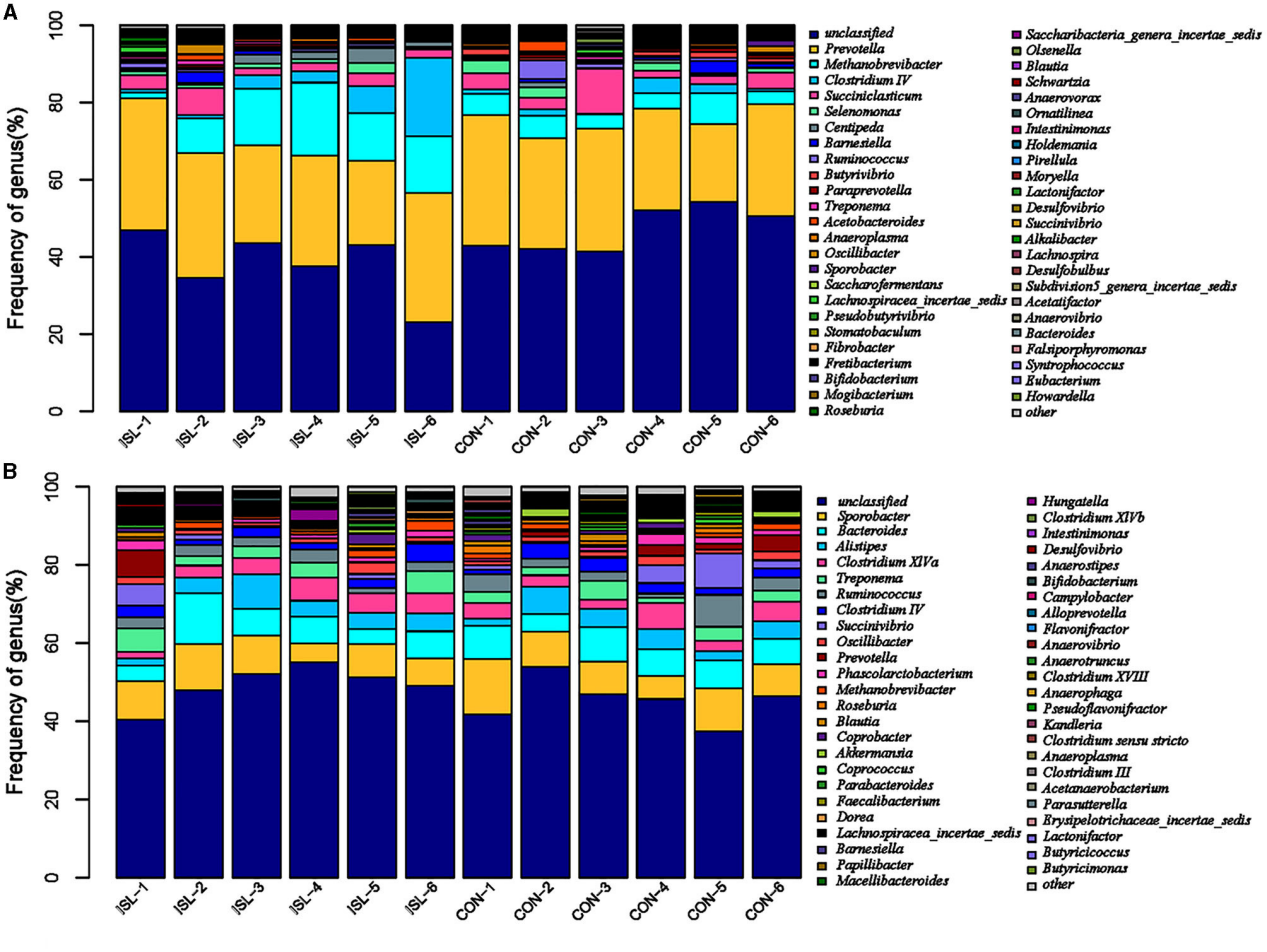
Distribution characteristics of horizontal microbiota in the ISL group group. **(A)** Distribution of rumen microbiota. **(B)** Distribution of fecal microbiota.

The rumen is the first stomach of ruminants, and rumen microorganisms are in dynamic equilibrium within the ruminant, maintaining normal growth of the body (27). Macrogenomic classification and sequencing of rumen microorganisms were conducted, and the rumen microbial population was analyzed based on genus level (Figure 3A). We detected Prevolella, Methanobrevibacter, Clostridium IV, Succiniclasticum, Selenomonas, and others between the ISL and CON groups. Among them, Prevolella, Methanobrevibacter, Succiniclasticum, Clostridium IV, and Selenomonas were the five dominant bacterial genera in the rumen of fattening sheep. Through further analysis of the rumen microbiota, significant changes were identified (accounting for 0.05% of the total sequence in at least one sample) (Table 4). A total of 6 genus level microbial changes were identified between the ISL group and the CON group, among which the relative abundance of Metanobrevibate and Centipeda in the ISL group was significantly upregulated compared to the CON group; the relative abundance of Butyrivibrio, Saccharoferments, Mogibacterium, and Pirellula was significantly downregulated (*P* < 0.05).

**Table 4:** Significant changes in the main microbial communities in the rumen of the ISL diet group.

**Phylum**	**Genus**	**Treatment**		**SEM**	***P*-value**
		**CON**	**ISL**		
Euryarchaeota	Methanobrevibacter	5.082^b^	11.813^a^	2.105	0.006
Firmicutes	Centipeda	0.447^b^	1.698^a^	0.466	0.017
Bacillota	Butyrivibrio	1.095^a^	0.393^b^	0.164	0.001
	Saccharofermentans	0.325^a^	0.178^b^	0.051	0.011
	Mogibacterium	0.182^a^	0.118^b^	0.022	0.011
Planctomycetota	Pirellula	0.162^a^	0.013^b^	0.064	0.034

^a,b^Values within a row with different superscripts differ significantly at *P* < 0.05.

### 3.4 Fecal microbiota

The fecal microbiota is closely related to the intestinal microbiota, among which the dominant microbiota is closely related to the balance of the intestinal microbiota, playing an important role in the structural function of the intestine, the digestion, absorption, and metabolism of nutrients, as well as the immune regulatory function of the body (28). Its changes affect the growth and development of animals. Based on genus level analysis of fecal microbial populations in the ISL and CON groups (Figure 3B), Sporobacter, Bacteroides, Alistipes, Clostridium_XIVa, and Treponema were detected between the ISL and CON groups. Among them, Sporobacter, Bacteroides, and Alistipes were the three dominant genera of fecal bacteria detected in the ISL and CON groups. In the ISL and CON groups of fecal microbiota, a total of 12 significantly changing microbiota were screened at the genus level (Table 5). In the ISL group, Papilibacter, Pseudoflavonifractor, Butyricicoccus, Anaerovorax, and Metanocorpusculum were significantly upregulated in relative abundance compared to the CON group; Compared to the CON group, the relative abundance of Roseburia, Coprococcus, Clostridium XVIII, Butyrivibrio, Parasutterella, Macelliberoides, and Porphyromonas was significantly reduced (*P* < 0.05).

**Table 5:** Change characteristics of the main microbial communities in the feces of the ISL diet group.

**Phylum**	**Genus**	**Treatment**		**SEM**	***P*-value**
		**CON**	**ISL**		
Bacillota	Roseburia	1.072^a^	0.655^b^	0.190	0.045
	Coprococcus	0.700^a^	0.338^b^	0.143	0.023
	Papillibacter	0.270^b^	0.472^a^	0.058	0.003
	Clostridium XVIII	0.227^a^	0.078^b^	0.065	0.039
	Pseudoflavonifractor	0.105^b^	0.182^a^	0.030	0.021
	Butyricicoccus	0.070^b^	0.122^a^	0.016	0.007
	Anaerovorax	0.028^b^	0.078^a^	0.017	0.011
	Butyrivibrio	0.068^a^	0.005^b^	0.025	0.021
Pseudomonadota	Parasutterella	0.148^a^	0.057^b^	0.026	0.003
Euryarchaeota	Methanocorpusculum	0.000^b^	0.147^a^	0.054	0.016
Bacteroidota	Macellibacteroides	0.525^a^	0.130^b^	0.161	0.027
	Porphyromonas	0.127^a^	0.012^b^	0.042	0.015

^a,b^Values within a row with different superscripts differ significantly at *P* < 0.05.

### 3.5 Rumen fluid metabolomics

Non-targeted LC-MS metabolomics analysis was conducted on 12 rumen fluid samples from fattening sheep in the ISL and CON groups, and differential metabolites were screened. A total of 164 differential metabolites were screened between the ISL and CON groups, including 62 anionic modes and 102 cationic modes (Supplementary Table S2).

**Figure 4:**
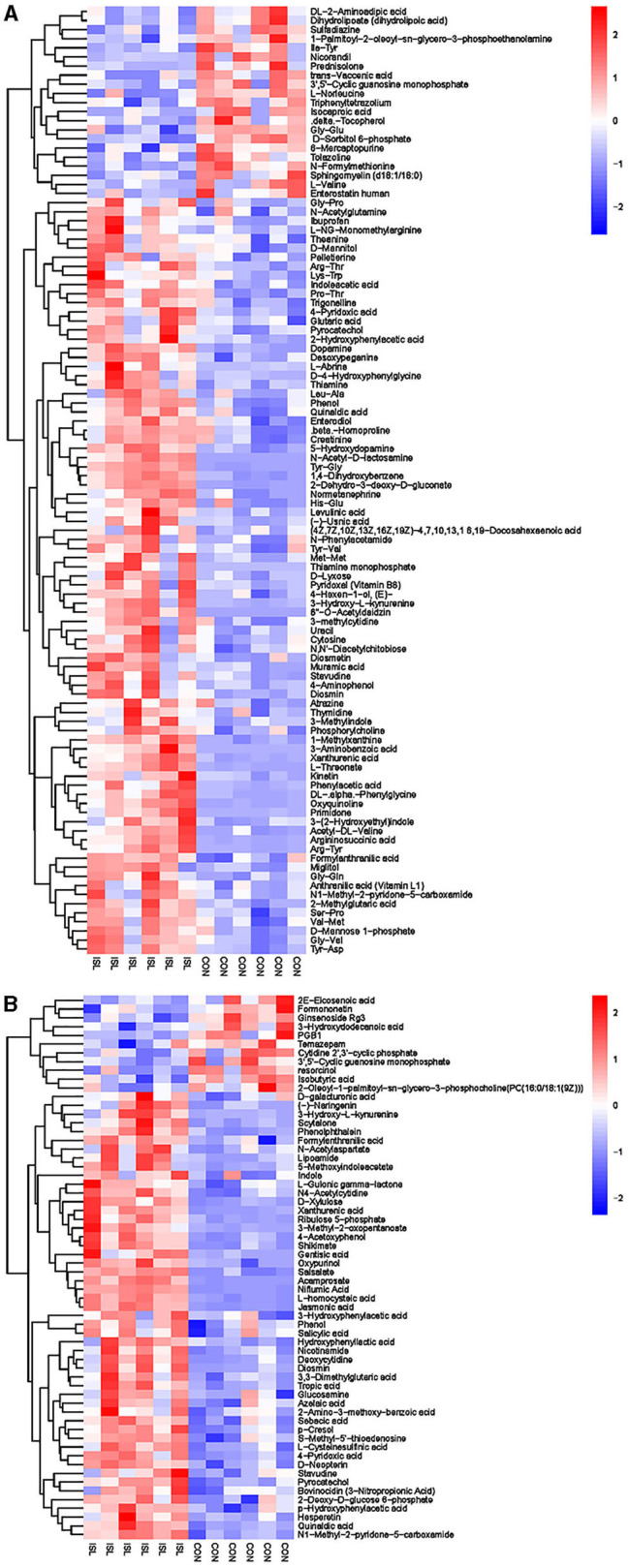
Hierarchical cluster analysis of rumen metabolites in fattening sheep. **(A)** Hierarchical clustering analysis of ISL groups in cationic mode. **(B)** Hierarchical cluster analysis of ISL group in anionic mode. The horizontal axis represents different experimental groups, the vertical axis represents the differential metabolites compared in the group, the blue color block represents the relative expression level of the corresponding position metabolites is down regulated, and the red color block represents the up regulation.

**Figure 5:**
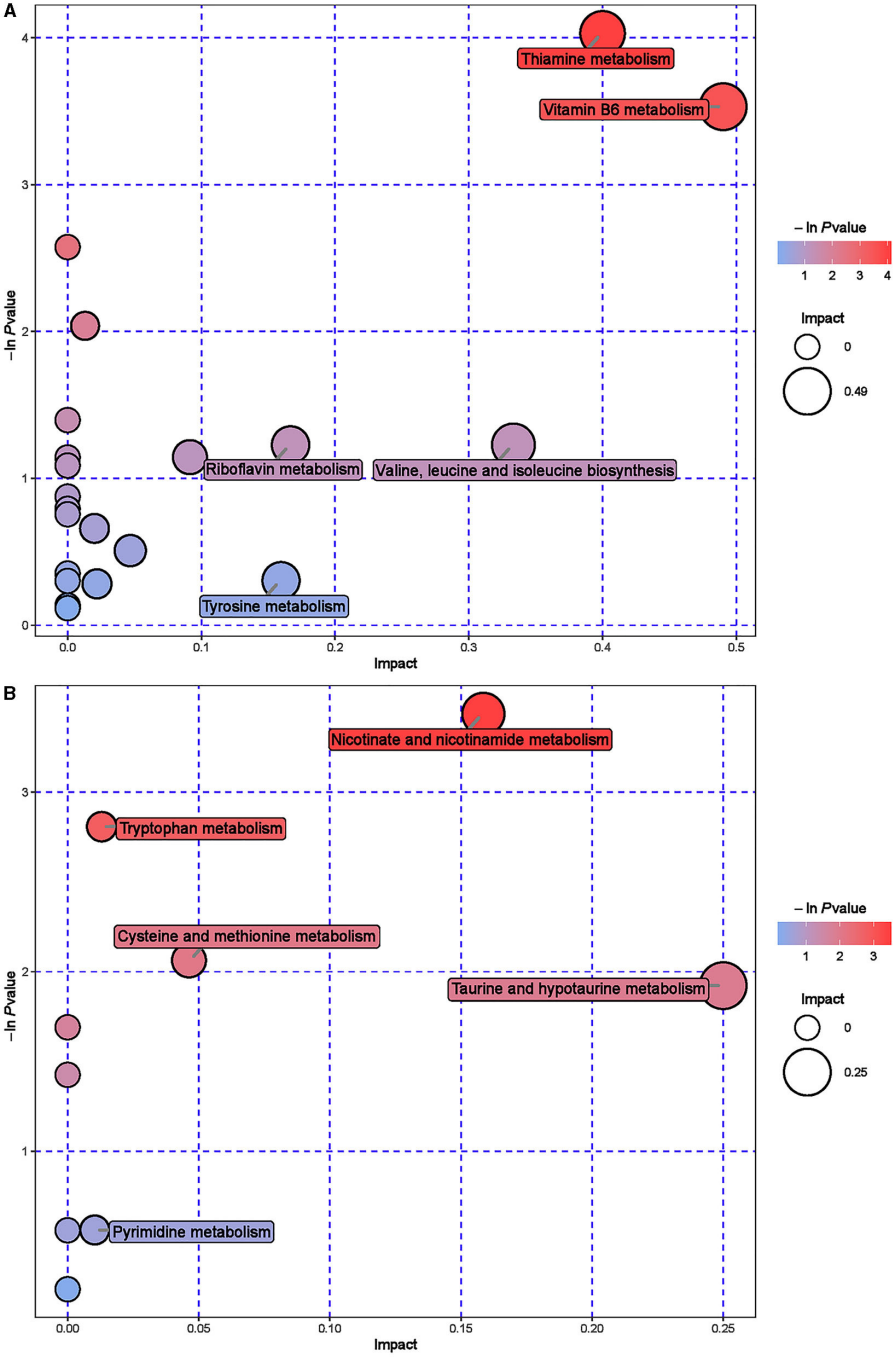
Pathway analysis of rumen metabolomics in fattening sheep. **(A)** Pathway analysis of ISL group in cationic mode. **(B)** Pathway analysis of ISL group in anionic mode. The color and size of bubbles indicate the impact of mint treatment on sample metabolism, while larger red bubbles indicate a greater impact on the pathway.

Further hierarchical clustering analysis was conducted on the differential metabolites between groups (Figure 4). The red color represents an upregulation of metabolites relative to the CON group, while the blue color represents a downregulation. The differences in different metabolites between the ISL group and the CON group were significant in the rumen fluid of fattening sheep. There were 32 types of differential metabolites significantly downregulated in the ISL group relative to the CON group, such as 2E-Eicosenoic acid, Formaronetin, DL-2-Aminoadipic acid, Dihydrolipoate (dihydrolipoic acid), and Sulfadiazine. A total of 132 differential metabolites were significantly upregulated, such as D-galacturonic acid, (-)-Naringenin, Gly-Pro, N-Acetylglutamine, and L-NG-Monomethylarginine. A total of 24 pathways with the highest correlation between rumen fluid metabolites were screened between the ISL group and the CON group (Supplementary Table S3, Figure 5), mainly including Thiamine metabolism, Nicotinate and nicotinamide metabolism, Vitamin B6 metabolism, Tryptophan metabolism, Taurine and hydroxyrine metabolism, and Cysteine and methionine metabolism.

### 3.6 Serum metabolomics

Serum is an important component of the blood, containing about 1,000 endogenous small molecule metabolites, mainly including plasma proteins, growth factors, hormones, inorganic ions, amino acids, glucose, nucleosides, lipids, and steroids (29). It is an important metabolomics research sample. Differential metabolite screening was conducted on serum samples. A total of 107 differential metabolites were screened between the ISL and CON groups, including 53 anionic and 54 cationic modes (Supplementary Table S4).

**Figure 6:**
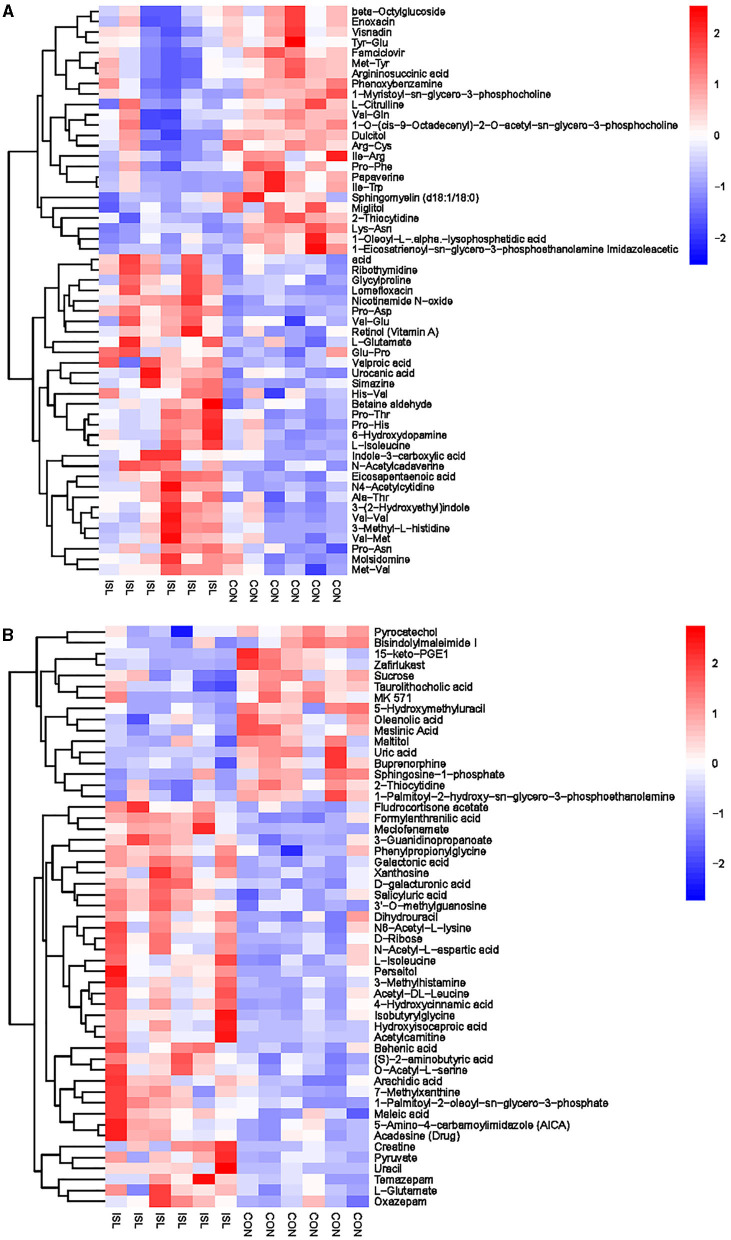
Hierarchical cluster analysis of serum metabolites in fattening sheep. **(A)** Hierarchical cluster analysis of ISL group serum in cationic mode. **(B)** Hierarchical cluster analysis of ISL group serum in anionic mode.

**Figure 7:**
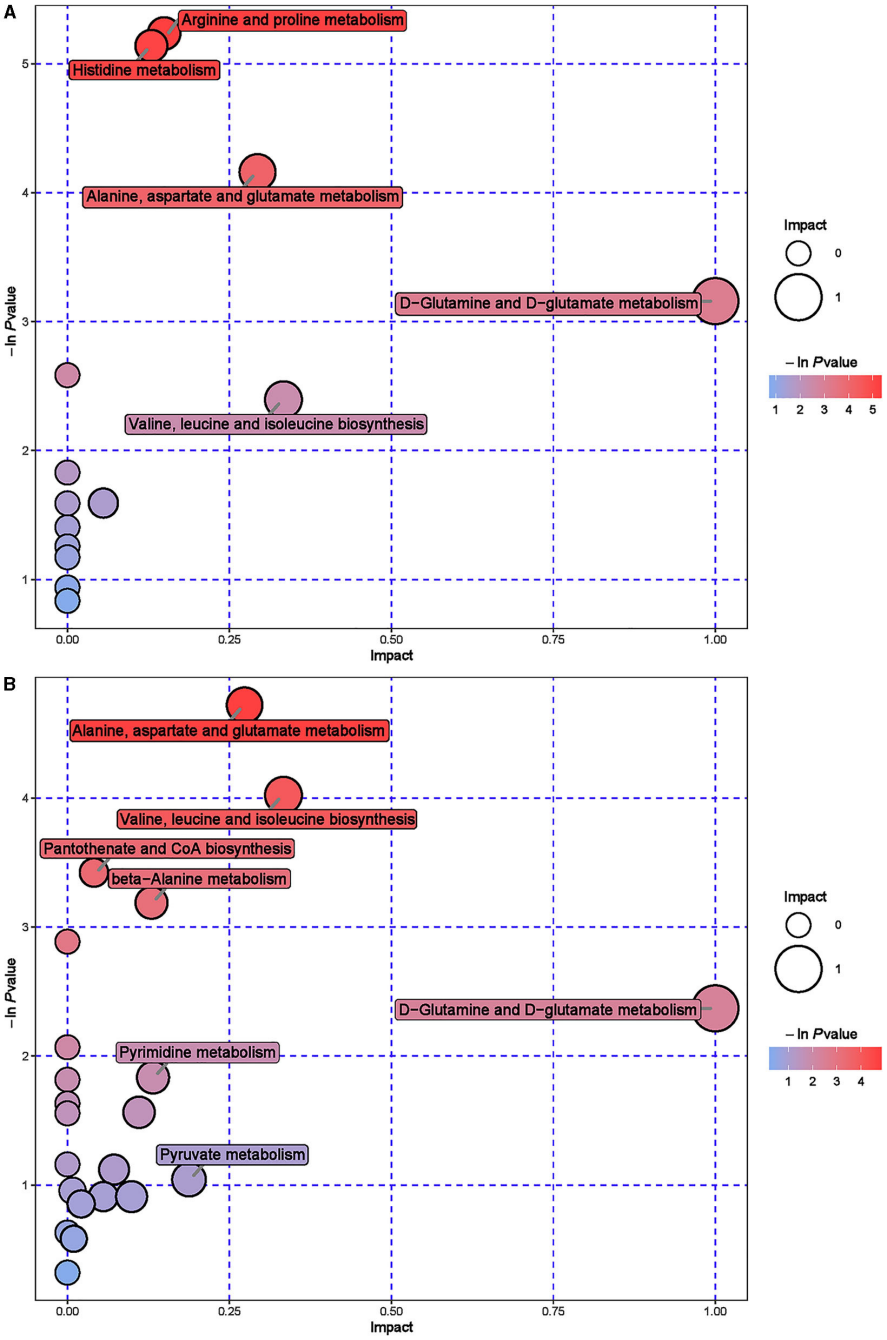
Pathway analysis of serum metabolomics. **(A)** Pathway analysis of ISL group serum in cationic mode. **(B)** Pathway analysis of ISL group serum under anionic mode.

The hierarchical cluster analysis of serum differential metabolites (Figure 6) showed that compared to the CON group, a total of 67 differential metabolites were significantly upregulated, such as Formylanthranilic acid, Meclofenamate, Flurocortisone acetate, Imidazoleacetic acid, Glycylproline, and Ribothymidine; a total of 40 differential metabolites were significantly downregulated, such as Pyrocatechol, Bisindolylmaleimide I, Zafirlukast, beta-Octylglucoside, Enoxacin, and Visnadin. Metabolic pathway analysis was conducted on the selected serum differential metabolites, and a total of 25 key metabolic pathways were identified (Supplementary Table S5, Figure 7). The key metabolic pathways affecting serum metabolism were mainly: Valine, leucine and isoleucine biosynthesis, Pantothenate and CoA biosynthesis, beta-Alanine metabolism, Arginine and proline metabolism, Histidine metabolism, Alanine, Aspartate and glucose metabolism, etc.

### 3.7 Urine metabolomics

Urine is the final excreta of metabolic products in the body (30). Analyzing changes in urine metabolites, key metabolic pathways, and potential biomarkers is of great significance for studying the mechanism of influence on the growth performance of feeding ISL fattening sheep. A total of 187 differential metabolites were screened between the ISL group and the CON group urine samples, of which 77 were screened for anionic mode and 110 were screened for cationic mode (Supplementary Table S6).

**Figure 8:**
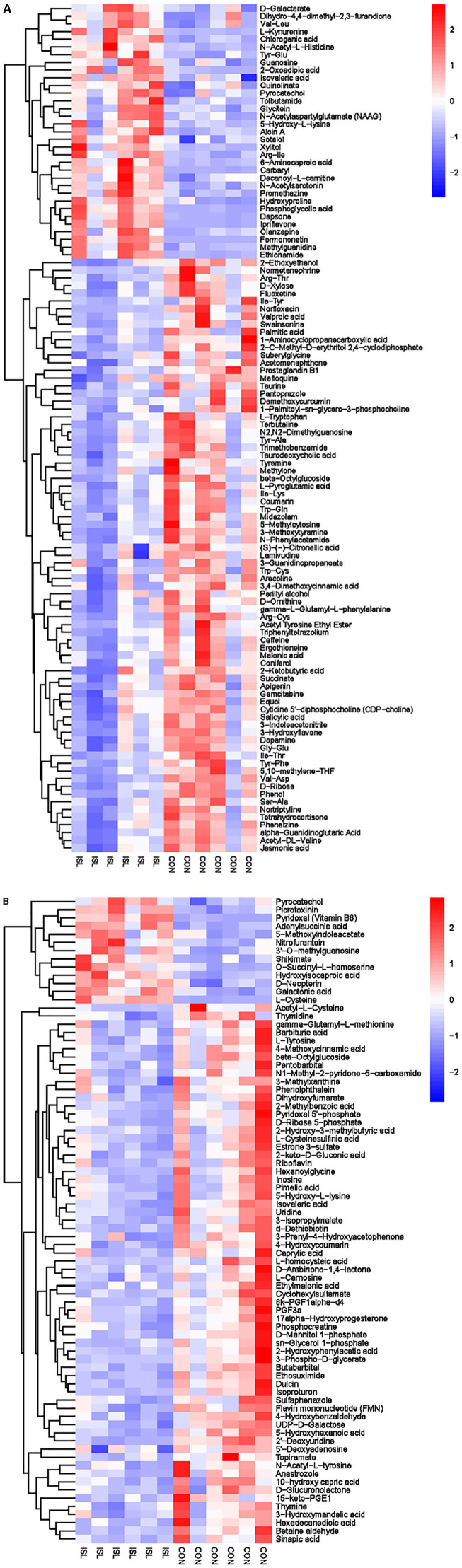
Hierarchical cluster analysis of urine metabolites in fattening sheep. **(A)** Hierarchical cluster analysis of ISL group urine in cationic mode. **(B)** Hierarchical cluster analysis of ISL group urine in anionic mode.

**Figure 9:**
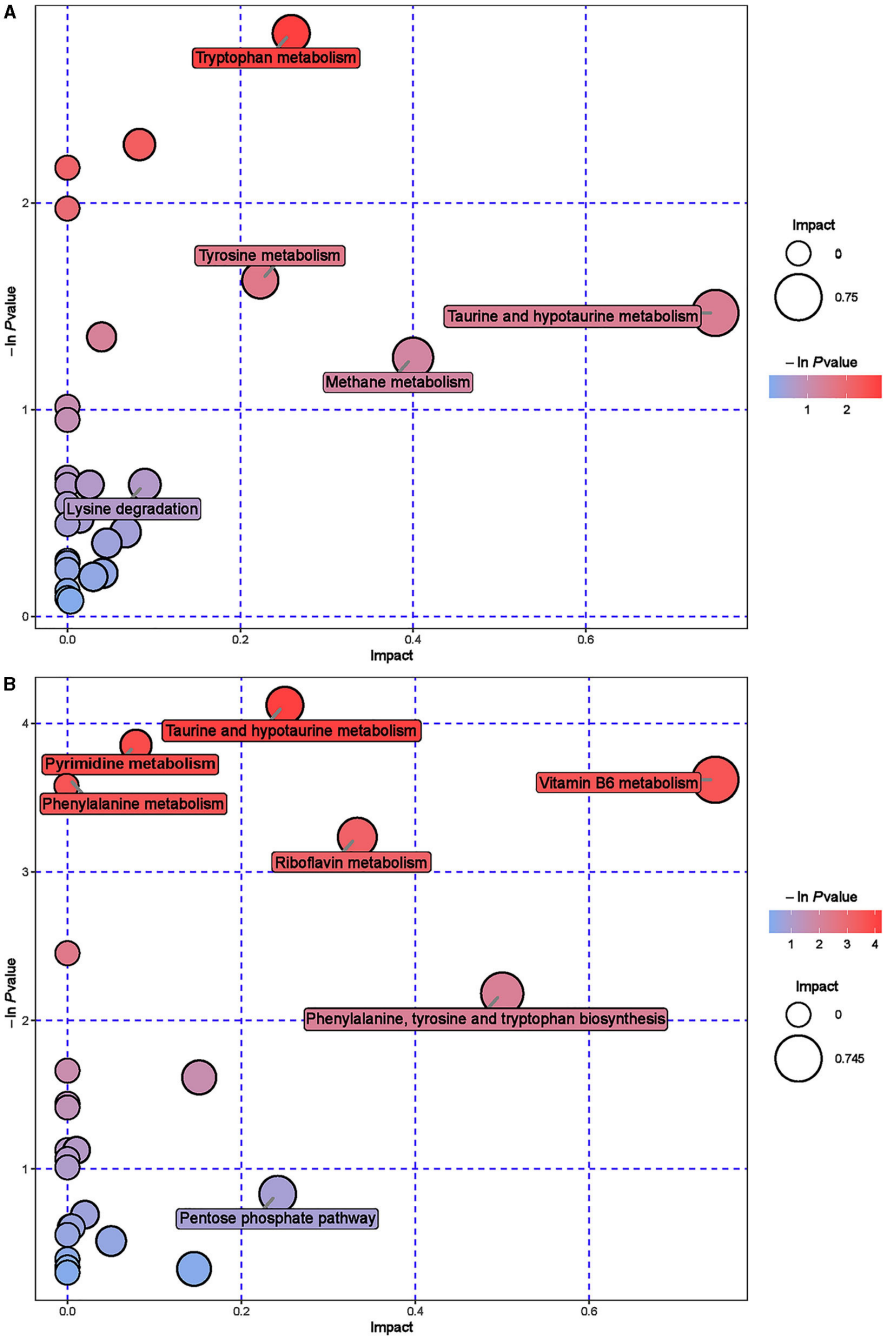
Pathway analysis of urine metabolomics. **(A)** Pathway analysis of ISL group urine in cationic mode. **(B)** Pathway analysis of ISL group urine under anionic mode.

Hierarchical cluster analysis was performed on the differential metabolites obtained from the analysis (Figure 8). Compared with the CON group, the ISL group significantly upregulated 46 differential metabolites in urine, such as Pyrocatechol, Picrotoxin, Pyridoxal (Vitamin B6), Guanosine, D-Galactalate, and Chlorogenic acid; A total of 141 differential metabolites were significantly downregulated, such as Acetyl-L-Cysteine, Thymidine, Barbituric acid, 2-Ethoxyethanol, Normanephrine, and Fluoxetine. A total of 42 key pathways with the highest correlation between urine samples metabolites in the ISL and CON groups were screened (Supplementary Table S7, Figure 9). The pathways with the highest correlation between feeding ISL on urine metabolites in fattening sheep were mainly Taurine and hydropotarine metabolism, Tryptophan metabolism, Pyrimidine metabolism, Phenylalanine metabolism, Riboflavin metabolism, and Vitamin B6 metabolism.

### 3.8 CircRNA bioinformatics

**Figure 10:**
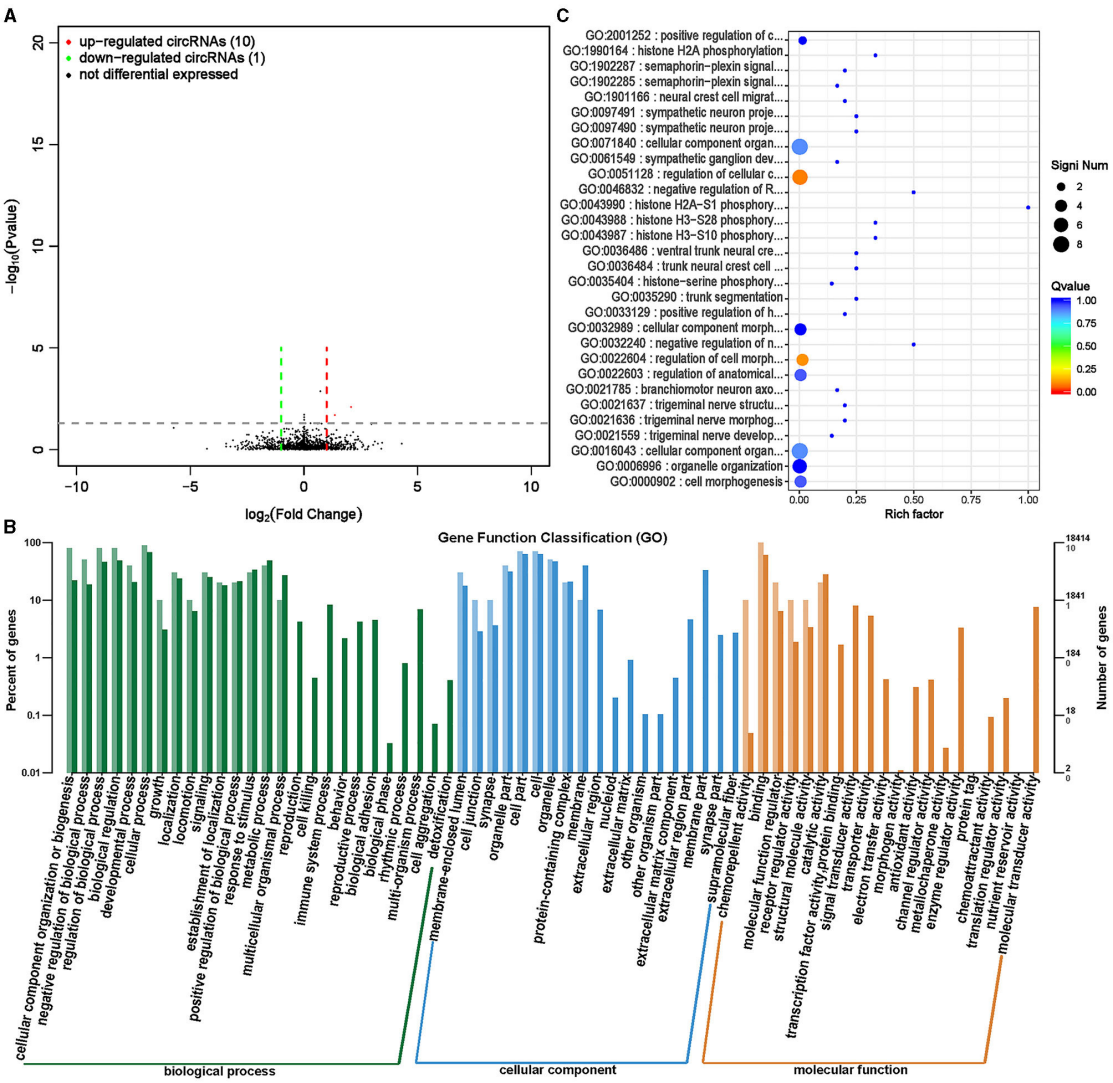
Differential expression analysis of circRNA between ISL group and CON group. **(A)** Volcano map of circRNA expression differences between ISL group and CON group. The horizontal axis represents the fold change [log (B/A)] value of the transcript expression difference between different groups, while the vertical axis represents the *P*-value of the transcript expression change. The smaller the *P*-value, the greater the log (*P*-value), and the more significant the difference. Red represents upregulated transcripts, green represents downregulated transcripts, and black represents non differential transcripts. **(B)** Histogram of host gene functional annotation classification for differentially expressed circRNA between ISL and CON groups. The horizontal axis represents the functional classification, while the vertical axis represents the number of genes within the classification (right) and their percentage in the total number of annotated genes (left). Light colors represent host genes, while dark colors represent all genes. **(C)** The top 30 functional scatter plots show significant enrichment of circRNA between the ISL group and the CON group. The vertical axis represents functional annotation information, while the horizontal axis represents the Rich factor corresponding to the function. The size of the Q-value is represented by the color of the dot. The smaller the Q-value, the closer the color is to red. The number of differentially expressed circRNA host genes is represented by the size of the dot.

CircRNA, as a special type of endogenous non coding RNA, is widely present in the cytoplasm of eukaryotic cells and plays an important regulatory role in biological growth and development (31, 32). A total of 11 significantly differentially expressed circRNAs were screened between the ISL and CON groups, of which 10 were significantly upregulated and 1 was significantly downregulated (Supplementary Table S8, Figure 10A).

For the screened differentially expressed circRNAs, further research was conducted on the distribution of their target genes in annotation function (Figure 10B). The differentially expressed cricRNA target genes mainly participate in biological processes such as cellular processes, biological process regulation, biological regulation, cell composition, tissue and biogenesis, and are mainly located at positions such as cells, cellular parts, organelles, and organelles. The main molecular functions are binding activity, catalytic activity, and molecular functional regulation. To further examine the function of focusing on differentially expressed circRNA target genes between the ISL group and the CON group, enrichment analysis was conducted on the target genes, and the top 30 functions with the highest enrichment degree were selected (Supplementary Table S9, Figure 10C). The circRNA target genes of fattening sheep fed with ISL were mainly enriched in the phosphorylation modification of regulating histones and the regulation of the nervous system. Discovery of alkaloids from the Isatis leaf with neuroprotective activity, which is consistent with the results of circRNA sequencing bioinformatics analysis (33, 34).

### 3.9 LncRNA bioinformatics

**Figure 11:**
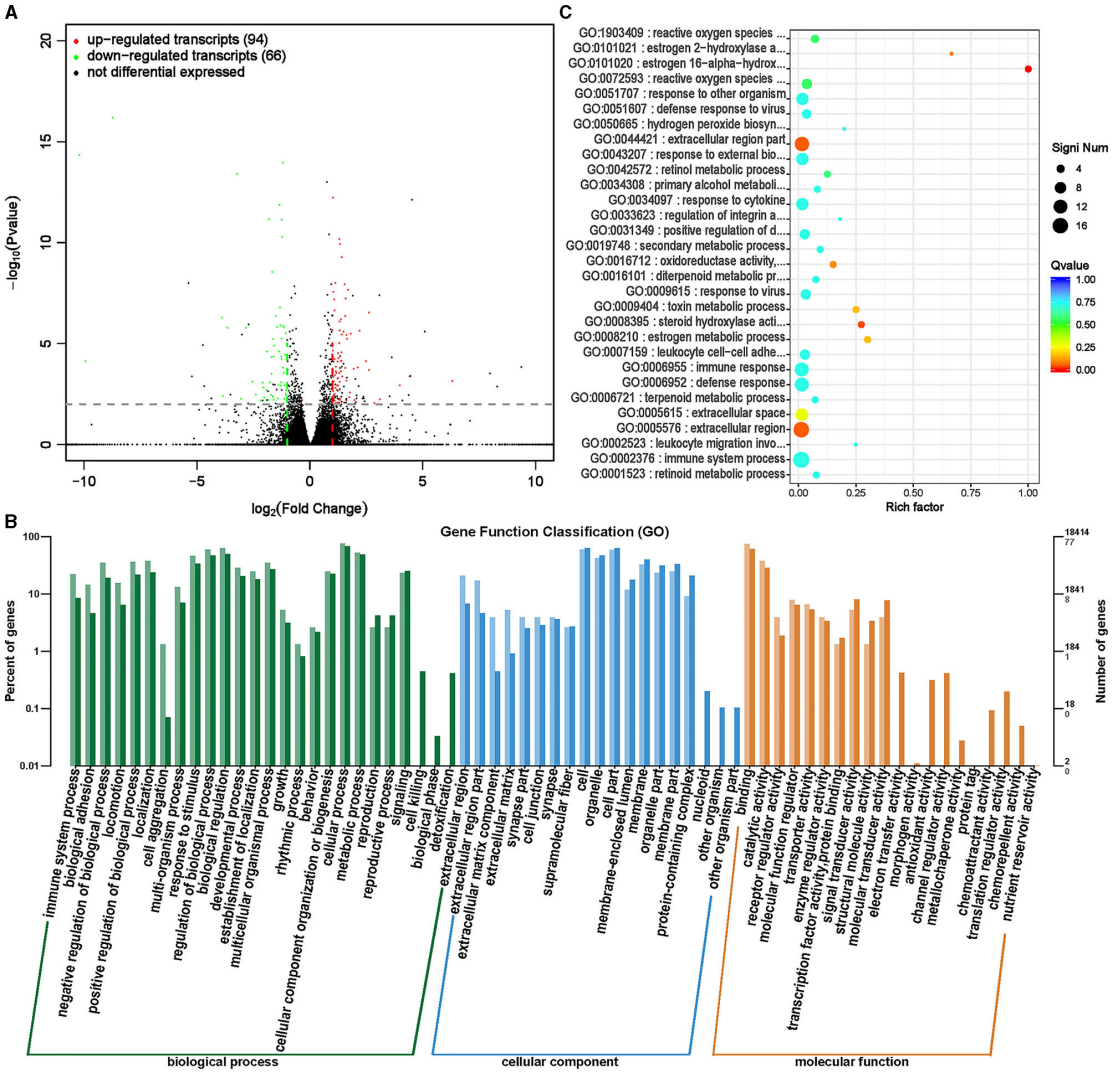
Differential expression analysis of transcriptome between ISL group and CON group. **(A)** Volcano map of transcript expression differences between ISL group and CON group. **(B)** Histogram of functional annotation classification of genes corresponding to ISL and CON differential transcripts. **(C)** The top 30 functional scatter plots show significant enrichment of differentially expressed transcripts in the ISL and CON groups.

LncRNAs are a type of RNAs with a length >200 nt and no protein encoding potential (35). LncRNA plays an important role in regulating growth and development, especially in regulating skeletal muscle growth and development (36). By analyzing the differential changes in lncRNA and its functional impact on target genes, it is of great significance to study the effects of ISL on the growth and development of fattening sheep. A total of 160 transcripts (lncRNA, mRNA) with significant differences were screened (Supplementary Table S10, Figure 11A), and 94 transcripts were significantly upregulated, including 64 lncRNAs and 30 mRNA, significantly downregulated 66 transcripts, 13 lncRNAs, and 53 mRNA.

The distribution analysis of the target genes of differential transcripts was conducted in the annotation function (Figure 11B). The target genes of transcripts mainly participate in biological processes such as cellular processes, metabolic processes, biological regulation, and biological regulation, mainly located in cells, organelles, and cellular parts. The main molecular functions focus on binding activity and catalytic activity. Enrichment analysis was conducted on the target genes of differential transcripts, and the top 30 functions with the highest enrichment degree were selected (Supplementary Table S11, Figure 11C). The target genes of differential transcripts are mainly enriched in immune regulatory functions, such as defense against viruses and immune responses. ISL has anti-inflammatory and antibacterial functions, and contains various antiviral substances, which can enhance the body's immunity and play an important role in regulating the health of fattening sheep (18, 19).

### 3.10 MiRNA bioinformatics

**Figure 12:**
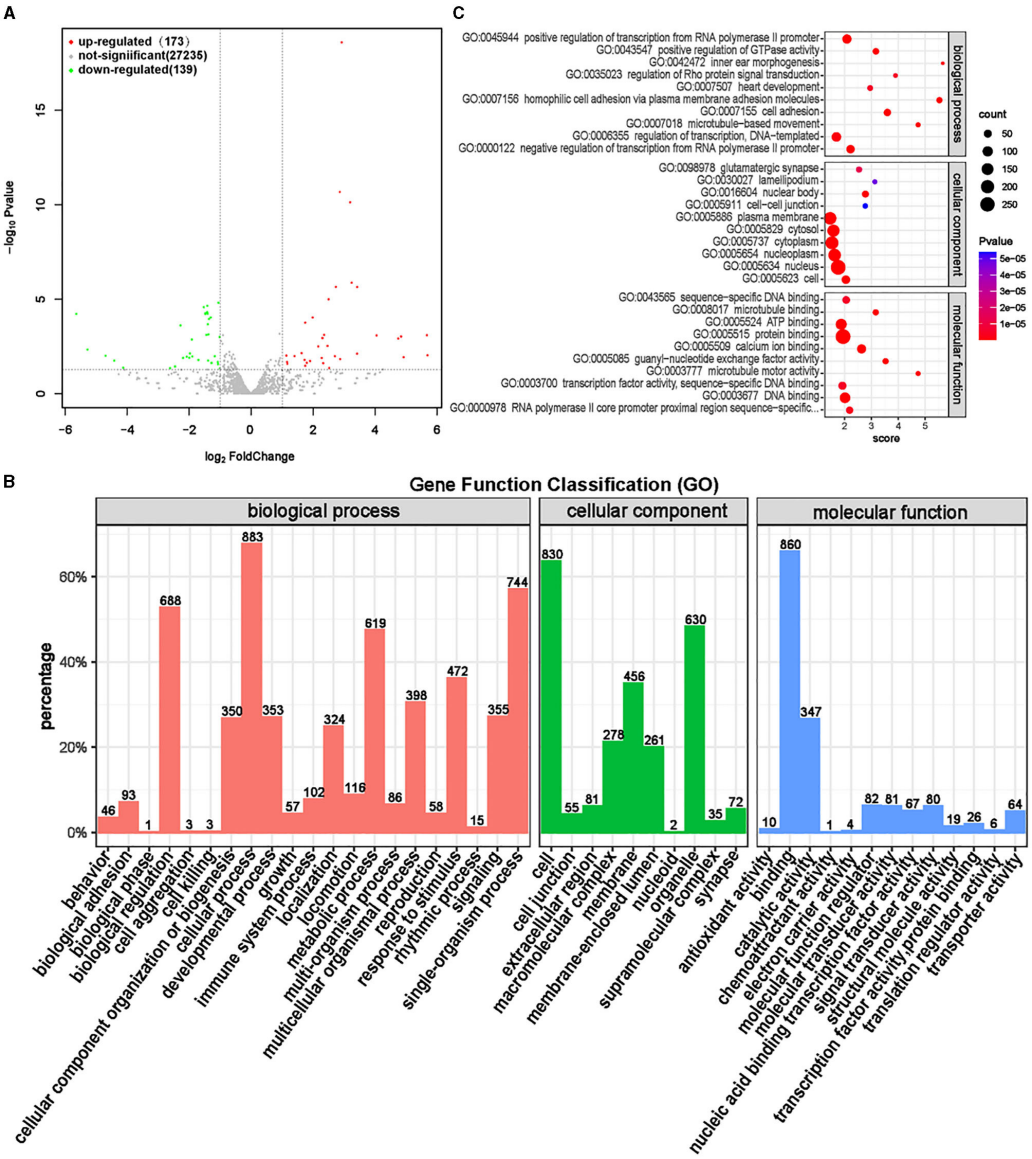
Differential expression analysis of miRNA between ISL group and CON group. **(A)** Volcano map of miRNA expression differences between ISL group and CON group. **(B)** ISL group and CON group differentially expressed miRNA GO annotation classification histogram. **(C)** The functional scatter plot shows a significant difference in miRNA enrichment between the ISL group and the CON group, with a top 10 * 3 degree (biological process, cellular component, and molecular function each with a top 10 degree).

MiRNAs are quite conserved in species evolution, and the expression of miRNAs found in animals has strict tissue specificity and timing (37). MiRNA plays various roles in cell growth and development, including regulating development, differentiation, apoptosis, and proliferation, and also plays a significant role in biological development (38). Differential expression analysis was conducted on miRNAs in the ISL and CON groups, and a total of 312 significantly different miRNAs were screened, of which 173 were significantly upregulated and 139 were significantly downregulated (Supplementary Table S12, Figure 12A).

Functional enrichment analysis was conducted on differentially targeted miRNAs obtained through screening, and gene functions of differentially targeted miRNAs were annotated and classified (Figure 12B). The differentially targeted miRNAs mainly participate in biological processes such as cellular processes, single biological processes, biological regulation, and metabolic processes, mainly located in cells, organelles, and membranes. The main molecular functions are focused on binding and catalytic activity. The top 10 functions with the highest enrichment in the three ontologies were selected (Supplementary Table S13, Figure 12C). The mRNA targeted by miRNAs is mainly enriched in transcription regulation and signal transduction functions, and the molecular function is mainly binding activity, including protein, calcium ions, DNA, and ATP. MiRNA bioinformatics analysis found that differential miRNAs have a regulatory effect on energy metabolism, thereby affecting the growth performance of fattening sheep fed with ISL.

## 4 Discusses

Most reports have shown that using Chinese herbal medicine as a feed additive in animal production can improve the growth performance of livestock and poultry (39, 40). As a plant rich in protein, minerals, and multiple vitamins, ISL is rich in amino acids such as phenylalanine and valine, which are essential amino acids in livestock (41). Therefore, adding an appropriate amount of ISL to livestock feed can enhance disease resistance and muscle performance.

In this study, after 2 months of feeding, it was found that the average daily gain of fattening sheep in the ISL group was significantly lower than that in the CON group (*P* < 0.05); the average daily feed intake of the ISL group decreased by 25% compared to the CON group. This may be due to the rich dietary fiber in the leaves, which are consumed by fattening sheep, leading to a stronger sense of fullness, reduced appetite, and reduced feed intake, resulting in a decrease in the average daily feed intake of the ISL group. A decrease in appetite and calorie intake in the diet can help control weight and reduce body fat rate, which may be the reason for the decrease in average daily weight gain in the ISL group. The rumen of ruminant animals utilizes microbial fermentation to degrade crude fibers in feed, and the mucosal muscle layers of the reticulum, omasum, and abomasum contract and relax, providing the power for digestion of crude feed. The more developed the muscle layer, the stronger the gastric digestion ability (42, 43). ISL contains relatively rich cellulose, which can promote gastrointestinal contraction and peristalsis, which may be the reason why the thickness of the reticulum and abomasum muscle layer in the ISL group is significantly higher than that in the CON group.

The appropriate concentration of ammonia nitrogen is the primary condition to ensure the efficiency of microbial protein synthesis (44). The protein levels in the diet of ISL were higher than those in the basal diet of the CON group, which may be the reason why the ammonia nitrogen levels in the ISL group were significantly higher than those in the CON group. Gastrointestinal microbiota can convert nitrogen sources in feed into bacterial proteins that can be absorbed and utilized by ruminants, playing an important role in animal metabolism and health. Analysis of rumen microbiota showed that compared to the CON group, the relative abundance of Metanobrevibate and Centipeda in the ISL group was significantly upregulated; the relative abundance of Butyrivibrio, Saccharofermentans, Mogibacterium, and Pirelula was significantly reduced. Methanobrevibater is the main hydrogen trophic methanogen in the rumen, which plays an important role in energy metabolism and adipose tissue deposition in animals. The abundance of methanogens is associated with lower body fat formation (45). Compared to forage, high starch diets can reduce rumen pH and inhibit the growth of methanogens, while the addition of ISL reduces the starch content of the diet, which may be the reason for the upregulation of relative abundance of Metanobrevibate (46). Butyrivibrio can induce the production of endotoxin, which can cause inflammatory reaction, metabolic and immune disorders, obesity, insulin resistance, diabetes, and other metabolic diseases, which is consistent with the fact that feeding Isatis leaf reduces the average daily weight gain and fat accumulation of fattening sheep (47, 48). Saccharoferments in the rumen microbiota can promote the production of low-density lipoprotein cholesterol (LDL-C), reduce the relative abundance of Saccharoferments, and lower blood lipids (49). Mogibacterium is usually abundant in the colon mucosa of human colorectal cancer patients, so an increase in its relative abundance may damage the health of goat rumen epithelium (50, 51). Analysis of fecal microbiota showed that compared to the CON group, the relative abundance of Papilibacter, Pseudoflavonifractor, Butyricicoccus, Anaerovorax, and Metanocorpusculum in the ISL group was significantly upregulated; Roseburia, Coprococcus, Clostridium XVIII, Butyrivibrio, Parasutterella, Macellibacteroides, and Porphyromonas were significantly downregulated. The relative abundance of Anaerotruncatus, Butyricoccus, and Papilibacter is positively correlated with the production of lipopolysaccharides (LPS). Butyricoccus can protect the intestinal barrier by producing short chain fatty acids (52); studies have found that high abundance of Parasutterella is associated with the activation of the human fatty acid synthesis pathway. In weight loss intervention trials, the abundance of Parasutterella was significantly reduced, and Parasutterella increased or was a mechanism for weight gain (53). In summary, it was found that the effect of feeding ISL on gastrointestinal microbiota mainly focuses on upregulating the beneficial genera for the body's immune system and downregulating the genera related to obesity. Therefore, ISL may affect growth performance by promoting the health of fattening sheep and reducing fat deposition.

To further explore the mechanism of the effect of adding ISL to the diet on the growth performance of fattening sheep, we conducted metabolomics analysis on rumen fluid, serum, and urine. Among them, 164 differential metabolites in rumen fluid were screened between the ISL and CON groups, with 24 key metabolic pathways; a total of 107 serum differential metabolites were screened, with 25 key metabolic pathways; there were 187 differential metabolites in urine and 42 key metabolic pathways. In the metabolism of rumen fluid, thiamine (vitamin B1) is an important component of co-carboxylase in glucose metabolism, mainly maintaining the normal metabolism of carbohydrates (54); niacin and niacinamide are two important members of the vitamin B family, playing important roles in metabolic processes, especially in fat and sugar metabolism; vitamin B6 is involved in the normal metabolism of sugars, proteins, and fats, and the regulation of these B-group vitamin metabolic pathways can promote the body's fat catabolism (55). In serum metabolism, beta-Alanine is produced through the metabolism of fat and glycogen, and plays an important role in cellular metabolism. In urine metabolism, taurine and taurine are involved in fat metabolism, helping the body utilize fat for energy supply while also regulating the immune system (56–58); Riboflavin (vitamin B2) is involved in energy metabolism in the body and is related to the metabolism of carbohydrates, proteins, nucleic acids, and fats (59). Based on the metabolomics analysis of rumen fluid, serum, and urine, the addition of ISL to the diet mainly regulates energy metabolism related pathways, especially fat metabolism. ISL can increase the consumption of body fat, reduce fat deposition, and thus lead to a decrease in the average daily weight gain of fattening sheep.

In addition, we also conducted transcriptomic analysis of rumen epithelial tissue to understand the changes in genes caused by feeding ISL at the transcriptome level, which helps us to gain a deeper understanding of the impact of ISL on the growth performance of fattening sheep. GO enrichment analysis of circRNA, lncRNA, and miRNA showed that these differential genes play important regulatory roles in normal cellular physiological processes, development, and immune regulation. It is of great significance for improving the physical health of fattening sheep.

The feeding experiment found that although feeding a diet containing ISL reduced the average daily gain of fattening sheep, it also reduced the average daily feed intake of fattening sheep. Based on the above analysis, the reason for the decrease in average daily feed intake of fattening sheep may be due to the rich cellulose content in the leaves (60), which creates a sense of satiety in fattening sheep. At the same time, the bitter taste of the ISL reduces the consumption of fattening sheep, leading to a decrease in feed intake.

Research has found that increasing dietary fiber can reduce fat deposition and improve lean meat percentage in the carcass (61–63); Feeding ISL can promote gastrointestinal health, increase the body's immunity and disease resistance. This study found that fattening sheep fed with a diet supplemented with ISL have a healthier body. The reason for the decrease in average daily weight gain may not only be due to ISL's ability to increase satiety and reduce food intake, but also due to ISL's ability to promote fat metabolism and reduce fat deposition. Although ISL reduces the average daily weight gain of fattening sheep, it can reduce the fat content of fattening sheep meat, which also has important application value for fattening sheep breeding.

## 5 Conclusion

In summary, the addition of ISL to the diet significantly increased the thickness of the reticulum and abomasum muscle layer in fattening sheep, slowed down the flow rate of feed at the end of the digestive tract, promoted further degradation of coarse feed, significantly increased ammonia nitrogen levels, and improved the composition of rumen microorganisms and intestinal microbiota. Compared to the CON group, the ISL group significantly increased the relative abundance of Methanobrevibacter and Centipeda in the rumen microbiota, while significantly reduced the relative abundance of Butyrivibrio, Saccharofermentans, Mogibacterium, and Pirellula; the relative abundance of intestinal microbiota Papilibacter, Pseudoflavonifractor, Butyricicoccus, Anaerovorax, and Methanocorpusculum was significantly upregulated; the relative abundance of Roseburia, Coprococcus, Clostridium XVIII, Butyrivibrio, Parasutterella, Macellibacteroides, and Porphyromonas significantly decreased. Metabolomics analysis of rumen fluid, serum, and urine showed that differential metabolites and differential metabolic pathways are mainly enriched in regulating energy metabolism, especially fat metabolism, to affect fat deposition and reduce fat rate in fattening sheep. Transcriptome analysis of rumen epithelial tissue revealed that whether circRNA, lncRNA, or miRNA, these differential genes play important regulatory roles in normal cellular physiological processes, development, and immune regulation. These results fully demonstrate that feeding fattening sheep with the addition of ISL diet has a positive effect on body health, improves the gastrointestinal microbiota of fattening sheep, enhances their immunity and resistance, promotes fat metabolism, and reduces fat accumulation in the body. ISL can improve the health level of fattening sheep and reduce fat, which is of great significance for healthy breeding of fattening sheep.

## Data availability statement

The original contributions presented in the study are publicly available. This data can be found here: https://www.ncbi.nlm.nih.gov/bioproject; PRJNA1051176, PRJNA1051169, and PRJNA1050748.

## Ethics statement

The animal studies were approved by the Animal Protection Committee of Anhui Agricultural University. The studies were conducted in accordance with the local legislation and institutional requirements. Written informed consent was obtained from the owners for the participation of their animals in this study.

## Author contributions

ZC: Conceptualization, Data curation, Writing—original draft. MY: Conceptualization, Formal analysis, Investigation, Writing—original draft. JZ: Conceptualization, Investigation, Writing—original draft. ZhZ: Investigation, Writing—original draft. ZL: Investigation, Writing—original draft. CY: Investigation, Writing—original draft. SS: Investigation, Writing—original draft. LW: Investigation, Writing—original draft. YL: Writing—review & editing. ZiZ: Funding acquisition, Writing—review & editing. HC: Funding acquisition, Writing—review & editing.
