# Supplementary material for: Multi-omics analysis on the mechanism of the effect of Isatis leaf on the growth performance of fattening sheep
Source: Front Vet Sci. 2024 Feb 7;11:1332457. doi: 10.3389/fvets.2024.1332457 (PMC10879442; doi:10.3389/fvets.2024.1332457)
Supplement: Supplementary file 1 [file Data_Sheet_1.docx]

Supplementary Material

**Multi-omics analysis on the mechanism of the effect of Isatis Leaf on the growth performance of fattening sheep**

**Mingliang Yi^1^****^†^, Zhikun Cao^1†^, Jialu Zhou^1†^, Zhiyu Zhang^1^, Zibo Liu^1^, Chao Yang^1^, Shixin Sun^1^, Lei Wang^1^, Yinghui Ling^1, 2^, Zijun Zhang^1, 2^, Hongguo Cao^1, 2^****^*^**

^*^**Correspondence:** Hongguo Cao:[caohongguo1@ahau.e](mailto:caohongguo1@126.com)du.cn

**Supplementary Tables and Figures**

**Table S1.** Effect of ISL on the morphology of intestinal segment in fattening sheep.

| Position | Item | CON (um) | ISL (um) |
| --- | --- | --- | --- |
| Duodenum | Muscularis | 431.17±3.29 | 470.95±21.25 |
|  | Villus | 383.11±5.00 | 434.42±18.84 |
| Jejunum | Muscularis | 272.98±5.24 | 240.90±18.99 |
|  | Villus | 362.08±38.72 | 332.07±9.98 |
| Ileum | Muscularis | 161.41±7.32 | 196.74±13.67 |
|  | Villus | 276.27 ±4.31 | 254.19±8.78 |
| Cecum | Muscularis mucosae | 344.66±11.46 | 347.61±16.33 |
|  | Mucosa | 396.35±14.37 | 351.91±8.49 |
| Colon | Muscularis mucosae | 276.68±12.00 | 245.73±1.31 |
|  | Mucosa | 535.91±23.65 | 501.3±7.94 |
| Rectum | Muscularis mucosae | 363.97±8.92 | 331.10±29.33 |
|  | Mucosa | 351.72±32.90 | 374.22±18.63 |

Note: * indicates a significant difference (P<0.05). ** indicates a highly significant difference (P<0.01).

**Table S2.** Significant differences in metabolites of rumen fluid between ISL and CON groups in fattening sheep.

| Name | MZ^1^ | R．T^2^（min） | VIP^3^ | Pvalue^4^ | FC^5^ |
| --- | --- | --- | --- | --- | --- |
| **Negative** | | | | | |
| Isobutyric acid | 87.046 | 106.772 | 1.941 | 0.005 | 0.746 |
| Phenol | 93.036 | 38.397 | 1.611 | 0.032 | 1.247 |
| p-Cresol | 107.050 | 45.147 | 2.156 | 0.001 | 1.709 |
| Pyrocatechol | 109.029 | 46.098 | 2.121 | 0.000 | 2.741 |
| Indole | 113.051 | 206.308 | 1.515 | 0.047 | 2.204 |
| 3-Methyl-2-oxopentanoate | 130.066 | 115.066 | 2.261 | 0.004 | 9.266 |
| Salicylic acid | 137.025 | 38.421 | 1.566 | 0.047 | 1.250 |
| D-Xylulose | 150.056 | 48.334 | 2.351 | 0.001 | 3.420 |
| 3-Hydroxyphenylacetic acid | 151.040 | 45.070 | 1.492 | 0.044 | 1.418 |
| p-Hydroxyphenylacetic acid | 151.040 | 171.694 | 1.742 | 0.003 | 1.495 |
| 4-Acetoxyphenol | 151.040 | 282.392 | 2.340 | 0.000 | 2.264 |
| L-Cysteinesulfinic acid | 152.002 | 68.174 | 2.096 | 0.001 | 1.505 |
| Oxypurinol | 152.035 | 44.457 | 2.021 | 0.000 | 2.763 |
| Gentisic acid | 153.019 | 68.628 | 2.256 | 0.010 | 4.463 |
| 3,3-Dimethylglutaric acid | 159.066 | 82.255 | 2.048 | 0.003 | 1.463 |
| Acamprosate | 162.019 | 44.679 | 2.474 | 0.000 | 31.899 |
| L-homocysteic acid | 164.002 | 125.912 | 2.450 | 0.000 | 4.825 |
| Formylanthranilic acid | 164.035 | 136.097 | 1.479 | 0.002 | 1.998 |
| N-Acetylaspartate | 174.040 | 78.775 | 1.549 | 0.033 | 2.029 |
| 4-Pyridoxic acid | 182.046 | 40.633 | 2.322 | 0.000 | 1.672 |
| Azelaic acid | 187.098 | 315.988 | 1.488 | 0.041 | 1.165 |
| D-galacturonic acid | 193.034 | 364.018 | 1.549 | 0.041 | 1.266 |
| Scytalone | 193.049 | 43.434 | 2.275 | 0.005 | 3.703 |
| Shikimate | 195.029 | 282.600 | 2.360 | 0.000 | 2.605 |
| Sebacic acid | 201.113 | 300.962 | 2.007 | 0.002 | 1.337 |
| Lipoamide | 204.052 | 105.380 | 1.738 | 0.023 | 3.109 |
| 5-Methoxyindoleacetate | 204.065 | 107.557 | 1.912 | 0.013 | 3.317 |
| 3-Hydroxy-L-kynurenine | 205.060 | 51.415 | 2.120 | 0.012 | 12.600 |
| 3-Hydroxydodecanoic acid | 215.164 | 107.522 | 1.522 | 0.025 | 0.779 |
| resorcinol | 219.065 | 33.583 | 2.007 | 0.006 | 0.719 |
| Xanthurenic acid | 221.056 | 120.423 | 2.430 | 0.001 | 4.347 |
| Tropic acid | 225.075 | 330.730 | 2.182 | 0.003 | 1.828 |
| Deoxycytidine | 227.091 | 187.352 | 2.097 | 0.002 | 1.688 |
| Quinaldic acid | 232.060 | 45.590 | 2.339 | 0.000 | 2.063 |
| Bovinocidin (3-Nitropropionic Acid) | 237.044 | 45.205 | 1.623 | 0.032 | 1.352 |
| L-Gulonic gamma-lactone | 237.061 | 149.055 | 1.872 | 0.005 | 1.493 |
| Glucosamine | 238.092 | 297.380 | 1.432 | 0.038 | 1.796 |
| Nicotinamide | 243.086 | 103.757 | 2.084 | 0.002 | 1.650 |
| Ribulose 5-phosphate | 246.039 | 45.515 | 2.362 | 0.002 | 4.719 |
| Jasmonic acid | 247.071 | 151.315 | 2.460 | 0.000 | 14.697 |
| 2-Deoxy-D-glucose 6-phosphate | 260.055 | 44.629 | 1.764 | 0.007 | 1.599 |
| Stavudine | 261.034 | 164.219 | 1.400 | 0.045 | 1.438 |
| Formononetin | 267.064 | 39.825 | 1.580 | 0.023 | 0.653 |
| D-Neopterin | 274.062 | 76.572 | 2.223 | 0.000 | 1.615 |
| Niflumic Acid | 281.055 | 106.464 | 2.507 | 0.000 | 7.134 |
| N4-Acetylcytidine | 284.088 | 158.201 | 2.112 | 0.000 | 2.190 |
| N1-Methyl-2-pyridone-5-carboxamide | 303.105 | 27.580 | 2.294 | 0.000 | 2.095 |
| Cytidine 2',3'-cyclic phosphate | 304.032 | 298.980 | 1.475 | 0.041 | 0.668 |
| 2E-Eicosenoic acid | 309.279 | 41.938 | 1.606 | 0.038 | 0.711 |
| Salsalate | 317.064 | 45.833 | 2.414 | 0.000 | 3.354 |
| Phenolphthalein | 317.083 | 24.873 | 2.293 | 0.001 | 2.407 |
| S-Methyl-5'-thioadenosine | 318.059 | 149.114 | 1.944 | 0.002 | 1.474 |
| Hesperetin | 323.053 | 113.784 | 2.051 | 0.012 | 1.788 |
| (-)-Naringenin | 331.080 | 44.577 | 2.095 | 0.011 | 2.439 |
| 2-Amino-3-methoxy-benzoic acid | 333.107 | 152.105 | 1.641 | 0.040 | 1.298 |
| PGB1 | 335.219 | 71.255 | 1.834 | 0.024 | 0.480 |
| 3',5'-Cyclic guanosine monophosphate | 344.037 | 299.566 | 1.528 | 0.024 | 0.619 |
| Temazepam | 359.075 | 45.125 | 1.375 | 0.043 | 0.684 |
| Hydroxyphenyllactic acid | 363.113 | 38.021 | 1.896 | 0.007 | 2.014 |
| Diosmin | 607.161 | 190.419 | 2.220 | 0.007 | 6.022 |
| 2-Oleoyl-1-palmitoyl-sn-glycero-3-phosphocholine(PC(16:0/18:1(9Z))) | 758.562 | 132.080 | 1.672 | 0.041 | 0.691 |
| Ginsenoside Rg3 | 783.487 | 144.285 | 1.854 | 0.002 | 0.579 |
| **Postive** | | | | | |
| 4-Aminophenol | 110.060 | 49.893 | 1.797 | 0.025 | 1.826 |
| Cytosine | 112.051 | 168.843 | 1.249 | 0.040 | 1.902 |
| Uracil | 113.035 | 156.939 | 1.334 | 0.046 | 1.522 |
| 3-Aminobenzoic acid | 120.045 | 43.681 | 2.302 | 0.008 | 10.132 |
| 1,4-Dihydroxybenzene | 128.071 | 79.520 | 2.287 | 0.000 | 2.317 |
| .beta.-Homoproline | 130.087 | 316.498 | 1.785 | 0.005 | 1.760 |
| Creatinine | 131.090 | 316.596 | 1.770 | 0.005 | 1.694 |
| 3-Methylindole | 132.081 | 39.423 | 1.492 | 0.043 | 1.737 |
| Anthranilic acid (Vitamin L1) | 138.055 | 269.393 | 1.556 | 0.022 | 1.433 |
| Pelletierine | 142.122 | 269.138 | 1.178 | 0.035 | 1.423 |
| DL-2-Aminoadipic acid | 144.065 | 280.845 | 1.662 | 0.024 | 0.563 |
| Oxyquinoline | 146.061 | 66.681 | 2.297 | 0.001 | 3.152 |
| D-Lyxose | 150.055 | 104.018 | 1.593 | 0.024 | 2.164 |
| Pyrocatechol | 152.071 | 266.695 | 1.648 | 0.015 | 1.225 |
| N-Acetylglutamine | 153.066 | 309.700 | 1.515 | 0.032 | 1.335 |
| N1-Methyl-2-pyridone-5-carboxamide | 153.066 | 89.475 | 1.551 | 0.029 | 1.585 |
| N-Phenylacetamide | 153.102 | 244.417 | 1.489 | 0.023 | 1.562 |
| Phenol | 158.060 | 92.392 | 2.022 | 0.000 | 1.676 |
| Isocaproic acid | 158.117 | 50.975 | 1.554 | 0.020 | 0.393 |
| L-Valine | 159.113 | 246.734 | 1.588 | 0.044 | 0.570 |
| Tolazoline | 160.097 | 95.304 | 1.432 | 0.022 | 0.791 |
| 1-Methylxanthine | 167.053 | 41.177 | 2.211 | 0.000 | 1.606 |
| Pyridoxal (Vitamin B6) | 168.065 | 104.494 | 1.395 | 0.046 | 1.613 |
| L-Norleucine | 173.126 | 258.615 | 1.728 | 0.003 | 0.389 |
| Quinaldic acid | 174.055 | 199.734 | 1.717 | 0.006 | 1.402 |
| 4-Pyridoxic acid | 184.061 | 41.190 | 2.018 | 0.002 | 1.484 |
| D-4-Hydroxyphenylglycine | 185.092 | 234.030 | 1.681 | 0.028 | 2.975 |
| 2-Methylglutaric acid | 188.092 | 323.008 | 2.058 | 0.001 | 1.558 |
| Gly-Pro | 190.123 | 38.356 | 1.610 | 0.036 | 1.931 |
| 2-Hydroxyphenylacetic acid | 194.081 | 312.105 | 1.745 | 0.030 | 1.544 |
| Dopamine | 195.112 | 230.480 | 2.248 | 0.000 | 2.906 |
| Glutaric acid | 196.060 | 343.502 | 1.471 | 0.044 | 1.157 |
| 6-Mercaptopurine | 196.983 | 164.072 | 1.518 | 0.023 | 0.711 |
| Kinetin | 198.076 | 309.692 | 1.936 | 0.017 | 1.473 |
| 3-(2-Hydroxyethyl)indole | 203.118 | 40.697 | 1.688 | 0.017 | 2.420 |
| Gly-Glu | 205.086 | 496.100 | 1.795 | 0.008 | 0.680 |
| Xanthurenic acid | 206.043 | 41.929 | 2.228 | 0.001 | 2.702 |
| 3-Hydroxy-L-kynurenine | 207.076 | 52.610 | 1.964 | 0.008 | 4.321 |
| Ibuprofen | 207.138 | 178.155 | 1.526 | 0.041 | 1.363 |
| Dihydrolipoate (dihydrolipoic acid) | 208.063 | 280.953 | 1.703 | 0.018 | 0.523 |
| Pro-Thr | 216.105 | 369.882 | 1.936 | 0.002 | 1.374 |
| Theanine | 216.134 | 444.482 | 1.398 | 0.045 | 1.294 |
| L-Abrine | 219.112 | 216.611 | 1.946 | 0.040 | 2.592 |
| Formylanthranilic acid | 226.069 | 286.825 | 1.796 | 0.006 | 1.365 |
| D-Sorbitol 6-phosphate | 227.137 | 87.732 | 2.068 | 0.000 | 0.729 |
| L-NG-Monomethylarginine | 230.157 | 286.131 | 1.621 | 0.028 | 1.801 |
| Levulinic acid | 233.100 | 81.710 | 1.951 | 0.022 | 2.690 |
| Desoxypeganine | 233.128 | 194.622 | 1.760 | 0.001 | 1.952 |
| Atrazine | 233.128 | 49.410 | 1.317 | 0.044 | 1.932 |
| Nicorandil | 234.052 | 365.345 | 1.490 | 0.035 | 0.227 |
| Indoleacetic acid | 236.091 | 345.116 | 1.681 | 0.007 | 1.303 |
| N-Formylmethionine | 238.070 | 180.435 | 1.966 | 0.001 | 0.600 |
| Gly-Val | 238.116 | 296.570 | 1.811 | 0.004 | 1.612 |
| 2-Dehydro-3-deoxy-D-gluconate | 239.082 | 108.519 | 2.324 | 0.000 | 4.244 |
| 4-Hexen-1-ol, (E)- | 239.139 | 348.869 | 1.787 | 0.004 | 1.582 |
| Leu-Ala | 241.089 | 365.630 | 1.440 | 0.037 | 2.270 |
| Thymidine | 243.097 | 102.313 | 1.361 | 0.041 | 1.806 |
| Ser-Pro | 244.128 | 302.649 | 1.823 | 0.002 | 1.438 |
| Miglitol | 246.079 | 156.911 | 1.461 | 0.009 | 1.826 |
| Normetanephrine | 247.107 | 193.803 | 1.980 | 0.001 | 2.836 |
| Gly-Gln | 248.064 | 173.779 | 1.933 | 0.003 | 2.427 |
| Arg-Thr | 258.152 | 351.277 | 1.769 | 0.015 | 1.320 |
| Primidone | 263.081 | 36.623 | 2.278 | 0.005 | 26.724 |
| Met-Met | 263.094 | 167.325 | 1.548 | 0.027 | 2.925 |
| Thiamine | 265.111 | 367.711 | 1.759 | 0.004 | 2.563 |
| Stavudine | 266.112 | 392.154 | 1.920 | 0.003 | 1.619 |
| Enterodiol | 267.139 | 193.387 | 1.748 | 0.005 | 1.715 |
| Sulfadiazine | 273.040 | 44.511 | 1.733 | 0.012 | 0.665 |
| Argininosuccinic acid | 273.123 | 222.519 | 2.307 | 0.002 | 9.131 |
| 3-methylcytidine | 275.138 | 268.023 | 1.417 | 0.027 | 1.369 |
| D-Mannose 1-phosphate | 278.063 | 316.058 | 1.799 | 0.006 | 1.600 |
| Triphenyltetrazolium | 282.133 | 327.415 | 1.385 | 0.030 | 0.897 |
| Tyr-Gly | 283.071 | 107.090 | 2.330 | 0.000 | 27.661 |
| trans-Vaccenic acid | 283.262 | 38.648 | 1.520 | 0.025 | 0.562 |
| Val-Met | 290.146 | 296.286 | 1.627 | 0.011 | 1.475 |
| Muramic acid | 293.133 | 314.041 | 1.754 | 0.016 | 2.879 |
| Trigonelline | 294.143 | 296.327 | 1.787 | 0.003 | 1.863 |
| Ile-Tyr | 294.154 | 269.507 | 1.647 | 0.010 | 0.610 |
| L-Threonate | 295.071 | 113.577 | 2.323 | 0.001 | 18.743 |
| Diosmetin | 301.070 | 44.517 | 1.325 | 0.032 | 1.775 |
| DL-.alpha.-Phenylglycine | 303.133 | 48.615 | 2.267 | 0.003 | 3.477 |
| Phenylacetic acid | 311.065 | 37.475 | 2.156 | 0.010 | 4.609 |
| Tyr-Val | 325.111 | 117.567 | 1.419 | 0.035 | 1.401 |
| (4Z,7Z,10Z,13Z,16Z,19Z)-4,7,10,13,1 6,19-Docosahexaenoic acid | 328.247 | 49.602 | 1.518 | 0.028 | 2.350 |
| Lys-Trp | 332.190 | 365.621 | 1.624 | 0.028 | 1.351 |
| Tyr-Asp | 338.131 | 313.025 | 2.059 | 0.000 | 1.931 |
| Prednisolone | 343.194 | 308.746 | 1.766 | 0.032 | 0.605 |
| (-)-Usnic acid | 345.095 | 46.119 | 1.807 | 0.024 | 1.974 |
| His-Glu | 345.143 | 108.395 | 1.930 | 0.006 | 2.455 |
| 3',5'-Cyclic guanosine monophosphate | 346.053 | 299.408 | 1.572 | 0.018 | 0.661 |
| Acetyl-DL-Valine | 357.142 | 310.171 | 2.112 | 0.001 | 4.512 |
| Arg-Tyr | 360.169 | 38.334 | 2.276 | 0.003 | 6.919 |
| 5-Hydroxydopamine | 361.137 | 51.006 | 2.135 | 0.000 | 2.522 |
| Thiamine monophosphate | 387.114 | 245.788 | 2.008 | 0.021 | 20.508 |
| .delta.-Tocopherol | 402.344 | 33.355 | 1.544 | 0.037 | 0.762 |
| D-Mannitol | 403.113 | 156.891 | 1.471 | 0.033 | 1.307 |
| 6''-O-Acetyldaidzin | 423.116 | 50.422 | 2.347 | 0.001 | 76.279 |
| N,N'-Diacetylchitobiose | 425.174 | 331.374 | 1.473 | 0.015 | 1.492 |
| N-Acetyl-D-lactosamine | 428.122 | 46.245 | 2.331 | 0.000 | 11.352 |
| Enterostatin human | 496.264 | 203.199 | 1.535 | 0.043 | 0.509 |
| Diosmin | 609.177 | 190.777 | 1.933 | 0.010 | 3.281 |
| 1-Palmitoyl-2-oleoyl-sn-glycero-3-phosphoethanolamine | 718.533 | 46.126 | 1.638 | 0.020 | 0.728 |

^1^ MZ = mass-to-charge ratio.

^2^ R.T = represents retention time.

^3^ VIP >1 and ^4^ Pvalue<0.05 are listed in the table. Pvalues were calculated according to Student’s T-test (n=6).

^5^ FC = fold change. If the fold change value is less than 1, it means that there is less metabolite in the ISL group than in the CON group.

**Table S3.** Pathway analysis of rumen fluid metabolomics in ISL and CON groups of fattening sheep.

| Pathway | Total | Hits^1)^ | Raw P^2)^ | Impact^3)^ | Hits compounds |
| --- | --- | --- | --- | --- | --- |
| Thiamine metabolism | 7 | 2 | 0.018 | 0.400 | Thiamine cpd:C00378; Thiamine monophosphate cpd:C01081 |
| Vitamin B6 metabolism | 9 | 2 | 0.029 | 0.490 | Pyridoxal cpd:C00250; 4-Pyridoxic acid cpd:C00847 |
| Pantothenate and CoA biosynthesis | 15 | 2 | 0.076 | 0.000 | L-Valine cpd:C00183; Uracil cpd:C00106 |
| Tryptophan metabolism | 41 | 3 | 0.130 | 0.013 | Formylanthranilic acid cpd:C05653; L-3-Hydroxykynurenine cpd:C03227; Indoleacetic acid cpd:C00954 |
| Phenylalanine metabolism | 9 | 1 | 0.247 | 0.000 | Ortho-Hydroxyphenylacetic acid cpd:C05852 |
| Riboflavin metabolism | 11 | 1 | 0.293 | 0.167 | Hydroquinone cpd:C00530 |
| Valine, leucine and isoleucine biosynthesis | 37 | 2 | 0.293 | 0.333 | L-Valine cpd:C00183 |
| Amino sugar and nucleotide sugar metabolism | 37 | 2 | 0.318 | 0.000 | Chitobiose cpd:C01674; D-Mannose 1-phosphate cpd:C00636 |
| Pyrimidine metabolism | 13 | 1 | 0.318 | 0.091 | Thymidine cpd:C00214; Uracil cpd:C00106 |
| Nicotinate and nicotinamide metabolism | 17 | 1 | 0.336 | 0.000 | N1-Methyl-2-pyridone-5-carboxamide cpd:C05842 |
| beta-Alanine metabolism | 17 | 1 | 0.415 | 0.000 | Uracil cpd:C00106 |
| Fructose and mannose metabolism | 19 | 1 | 0.452 | 0.000 | D-Mannose 1-phosphate cpd:C00636 |
| Lysine degradation | 20 | 1 | 0.469 | 0.000 | Aminoadipic acid cpd:C00956 |
| Alanine, aspartate and glutamate metabolism | 23 | 1 | 0.517 | 0.020 | Argininosuccinic acid cpd:C03406 |
| Glycerophospholipid metabolism | 29 | 1 | 0.602 | 0.047 | Phosphorylcholine cpd:C00588 |
| Valine, leucine and isoleucine degradation | 38 | 1 | 0.702 | 0.000 | L-Valine cpd:C00183 |
| Biosynthesis of unsaturated fatty acids | 42 | 1 | 0.738 | 0.000 | (4Z,7Z,10Z,13Z,16Z,19Z)-Docosahexaenoic acid cpd:C06429 |
| Tyrosine metabolism | 42 | 1 | 0.738 | 0.160 | Dopamine cpd:C03758 |
| Arginine and proline metabolism | 44 | 1 | 0.754 | 0.022 | Argininosuccinic acid cpd:C03406 |
| Aminoacyl-tRNA biosynthesis | 64 | 1 | 0.872 | 0.000 | L-Valine cpd:C00183 |
| Purine metabolism | 68 | 1 | 0.888 | 0.000 | Cyclic GMP cpd:C00942 |
| Cysteine and methionine metabolism | 28 | 2 | 0.127 | 0.046 | 5'-Methylthioadenosine cpd:C00170; 3-Sulfinoalanine cpd:C00606 |
| Taurine and hypotaurine metabolism | 7 | 1 | 0.146 | 0.250 | 3-Sulfinoalanine cpd:C00606 |
| Ascorbate and aldarate metabolism | 9 | 1 | 0.184 | 0.000 | L-Gulonolactone cpd:C01040 |

^1^ Hits represent the number of significantly different ruminal metabolites matched in one pathway.

^2^ P is the original P value obtained by pathway analysis.

^3^ Impact is the influencing factor of the pathway obtained by topology analysis.

**Table S4.** Significant differences in metabolites between ISL group and CON group in serum of fattening sheep.

| Name | MZ | R.T(min) | VIP | Pvalue | FC |
| --- | --- | --- | --- | --- | --- |
| **Negative** | | | | | |
| Pyruvate | 87.009 | 121.770 | 1.415 | 0.045 | 1.750 |
| (S)-2-aminobutyric acid | 102.056 | 370.967 | 2.015 | 0.001 | 1.390 |
| Uracil | 111.019 | 88.176 | 1.966 | 0.031 | 3.020 |
| 5-Amino-4-carbamoylimidazole (AICA) | 125.046 | 118.720 | 1.658 | 0.027 | 1.712 |
| O-Acetyl-L-serine | 128.035 | 370.754 | 1.714 | 0.013 | 1.272 |
| Creatine | 130.065 | 245.772 | 1.370 | 0.045 | 13.777 |
| L-Isoleucine | 130.087 | 262.957 | 1.499 | 0.031 | 1.377 |
| Hydroxyisocaproic acid | 131.071 | 124.326 | 1.728 | 0.045 | 2.608 |
| Isobutyrylglycine | 144.066 | 194.273 | 1.786 | 0.025 | 2.661 |
| L-Glutamate | 146.045 | 293.426 | 1.333 | 0.041 | 1.280 |
| 3-Methylhistamine | 146.072 | 48.779 | 1.941 | 0.025 | 5.495 |
| 7-Methylxanthine | 147.029 | 366.755 | 1.645 | 0.025 | 1.320 |
| D-Ribose | 149.045 | 349.900 | 1.754 | 0.012 | 1.969 |
| 3-Guanidinopropanoate | 152.043 | 334.417 | 1.889 | 0.004 | 1.229 |
| 5-Hydroxymethyluracil | 158.060 | 139.826 | 1.566 | 0.020 | 0.753 |
| Formylanthranilic acid | 164.035 | 68.879 | 1.970 | 0.001 | 1.790 |
| Uric acid | 167.020 | 302.345 | 1.828 | 0.028 | 0.186 |
| Acetyl-DL-Leucine | 172.097 | 177.472 | 1.791 | 0.014 | 2.309 |
| Dihydrouracil | 173.056 | 291.849 | 1.525 | 0.047 | 1.738 |
| N-Acetyl-L-aspartic acid | 174.040 | 361.337 | 1.671 | 0.021 | 1.679 |
| Maleic acid | 175.024 | 412.632 | 1.633 | 0.015 | 1.217 |
| 4-Hydroxycinnamic acid | 185.019 | 22.242 | 1.770 | 0.011 | 3.232 |
| N6-Acetyl-L-lysine | 187.108 | 335.488 | 1.608 | 0.020 | 1.268 |
| D-galacturonic acid | 193.035 | 362.862 | 2.007 | 0.002 | 1.288 |
| Salicyluric acid | 194.045 | 156.787 | 1.667 | 0.009 | 1.307 |
| Galactonic acid | 195.050 | 368.430 | 1.990 | 0.001 | 1.443 |
| Acetylcarnitine | 202.108 | 167.355 | 1.925 | 0.040 | 4.095 |
| Phenylpropionylglycine | 206.082 | 167.564 | 1.549 | 0.019 | 1.264 |
| Perseitol | 212.090 | 251.512 | 1.744 | 0.012 | 1.297 |
| Pyrocatechol | 219.065 | 34.008 | 1.332 | 0.011 | 0.609 |
| Acadesine (Drug) | 239.078 | 118.727 | 1.666 | 0.026 | 1.947 |
| 2-Thiocytidine | 258.055 | 169.707 | 1.924 | 0.002 | 0.624 |
| Xanthosine | 283.067 | 288.178 | 1.733 | 0.020 | 1.571 |
| Meclofenamate | 294.007 | 35.512 | 2.251 | 0.005 | 9.592 |
| 3'-O-methylguanosine | 296.099 | 189.024 | 1.959 | 0.001 | 1.252 |
| Oxazepam | 307.023 | 93.835 | 1.628 | 0.022 | 1.918 |
| Arachidic acid | 311.295 | 40.774 | 1.538 | 0.026 | 1.275 |
| Temazepam | 321.039 | 68.071 | 1.524 | 0.044 | 2.165 |
| Behenic acid | 339.326 | 40.951 | 1.604 | 0.021 | 1.306 |
| Sucrose | 342.118 | 315.467 | 1.425 | 0.031 | 0.591 |
| Maltitol | 344.133 | 260.680 | 1.477 | 0.029 | 0.627 |
| 15-keto-PGE1 | 351.221 | 38.243 | 1.627 | 0.035 | 0.235 |
| Fludrocortisone acetate | 403.195 | 259.111 | 1.839 | 0.007 | 1.663 |
| Bisindolylmaleimide I | 411.181 | 32.637 | 1.531 | 0.026 | 0.771 |
| Sphingosine-1-phosphate | 438.260 | 188.282 | 1.531 | 0.044 | 0.635 |
| 1-Palmitoyl-2-hydroxy-sn-glycero-3-phosphoethanolamine | 452.276 | 185.396 | 1.706 | 0.014 | 0.819 |
| Oleanolic acid | 455.350 | 43.723 | 1.572 | 0.016 | 0.723 |
| Buprenorphine | 466.291 | 182.203 | 1.839 | 0.006 | 0.805 |
| Maslinic Acid | 471.345 | 48.134 | 1.842 | 0.006 | 0.721 |
| Taurolithocholic acid | 482.294 | 46.745 | 1.490 | 0.011 | 0.631 |
| MK 571 | 573.126 | 333.185 | 1.735 | 0.034 | 0.395 |
| Zafirlukast | 574.188 | 38.052 | 1.994 | 0.010 | 0.194 |
| 1-Palmitoyl-2-oleoyl-sn-glycero-3-phosphate | 673.477 | 124.828 | 1.666 | 0.015 | 1.344 |
| **Postive** | | | | | |
| Betaine aldehyde | 102.090 | 343.589 | 1.874 | 0.028 | 1.213 |
| Imidazoleacetic acid | 127.049 | 328.590 | 1.587 | 0.027 | 1.295 |
| Nicotinamide N-oxide | 139.049 | 150.672 | 2.190 | 0.001 | 1.927 |
| Urocanic acid | 139.049 | 267.843 | 1.690 | 0.029 | 1.421 |
| L-Glutamate | 148.060 | 370.703 | 1.800 | 0.030 | 1.248 |
| 3-Methyl-L-histidine | 152.080 | 47.578 | 1.964 | 0.031 | 3.877 |
| 6-Hydroxydopamine | 170.080 | 240.592 | 1.932 | 0.027 | 1.854 |
| Glycylproline | 173.091 | 367.356 | 2.002 | 0007 | 1.699 |
| L-Isoleucine | 173.127 | 229.461 | 2.021 | 0.018 | 1.835 |
| L-Citrulline | 176.102 | 393.110 | 1.670 | 0.049 | 0.809 |
| Pro-Thr | 181.096 | 143.921 | 1.981 | 0.020 | 2.597 |
| Valproic acid | 186.147 | 37.645 | 1.455 | 0.039 | 1.612 |
| Indole-3-carboxylic acid | 203.080 | 189.372 | 1.847 | 0.035 | 2.272 |
| 3-(2-Hydroxyethyl)indole | 206.052 | 294.143 | 1.907 | 0.027 | 2.143 |
| Molsidomine | 208.098 | 38.396 | 1.858 | 0.030 | 1.422 |
| Ala-Thr | 208.132 | 41.254 | 2.036 | 0.006 | 1.703 |
| N-Acetylcadaverine | 211.081 | 184.309 | 1.834 | 0.010 | 1.614 |
| Met-Val | 213.105 | 277.738 | 1.829 | 0.018 | 1.664 |
| Val-Val | 217.153 | 261.362 | 1.842 | 0.041 | 2.899 |
| Simazine | 219.115 | 99.908 | 2.007 | 0.005 | 1.907 |
| Dulcitol | 224.112 | 283.397 | 1.788 | 0.018 | 0.547 |
| Glu-Pro | 227.101 | 268.535 | 1.672 | 0.033 | 1.160 |
| Pro-His | 235.118 | 139.404 | 1.889 | 0.039 | 1.869 |
| Pro-Phe | 245.123 | 292.865 | 1.603 | 0.031 | 0.652 |
| Val-Gln | 246.144 | 313.231 | 1.416 | 0.036 | 0.593 |
| Val-Glu | 247.128 | 354.107 | 1.411 | 0.038 | 1.609 |
| Pro-Asn | 247.139 | 338.133 | 1.738 | 0.030 | 1.506 |
| Miglitol | 249.148 | 187.303 | 1.957 | 0.009 | 0.686 |
| His-Val | 254.131 | 249.039 | 1.633 | 0.040 | 1.437 |
| Ribothymidine | 259.091 | 328.566 | 1.520 | 0.031 | 1.366 |
| 2-Thiocytidine | 260.069 | 169.876 | 2.063 | 0.005 | 0.661 |
| Eicosapentaenoic acid | 285.220 | 34.142 | 2.185 | 0.003 | 1.826 |
| N4-Acetylcytidine | 286.102 | 158.142 | 2.085 | 0.018 | 2.408 |
| Retinol (Vitamin A) | 286.227 | 34.142 | 1.933 | 0.019 | 1.790 |
| Ile-Arg | 288.202 | 323.000 | 1.804 | 0.015 | 0.729 |
| Lys-Asn | 305.119 | 326.400 | 2.024 | 0.005 | 0.643 |
| Val-Met | 309.154 | 240.242 | 1.769 | 0.048 | 2.051 |
| Met-Tyr | 312.110 | 306.241 | 1.683 | 0.018 | 0.587 |
| Argininosuccinic acid | 313.113 | 306.241 | 1.692 | 0.019 | 0.636 |
| beta-Octylglucoside | 337.159 | 354.300 | 1.469 | 0.034 | 0.671 |
| Arg-Cys | 338.154 | 393.075 | 1.800 | 0.009 | 0.546 |
| Enoxacin | 338.162 | 353.817 | 1.563 | 0.024 | 0.681 |
| Papaverine | 339.154 | 193.098 | 1.699 | 0.021 | 0.275 |
| Ile-Trp | 340.156 | 193.325 | 1.658 | 0.024 | 0.382 |
| Famciclovir | 344.133 | 315.326 | 1.678 | 0.012 | 0.523 |
| Lomefloxacin | 352.146 | 295.811 | 2.256 | 0.005 | 1.807 |
| Visnadin | 353.132 | 293.863 | 1.601 | 0.027 | 0.633 |
| Phenoxybenzamine | 367.148 | 282.726 | 1.511 | 0.042 | 0.576 |
| Tyr-Glu | 371.143 | 294.147 | 1.592 | 0.040 | 0.575 |
| 1-Myristoyl-sn-glycero-3-phosphocholine | 468.307 | 183.904 | 1.788 | 0.013 | 0.623 |
| 1-Oleoyl-L-.alpha.-lysophosphatidic acid | 478.291 | 184.670 | 1.827 | 0.024 | 0.635 |
| 1-Eicosatrienoyl-sn-glycero-3-phosphoethanolamine | 504.304 | 182.619 | 2.132 | 0.016 | 0.816 |
| 1-O-(cis-9-Octadecenyl)-2-O-acetyl-sn-glycero-3-phosphocholine | 550.383 | 170.834 | 1.574 | 0.033 | 0.656 |
| Sphingomyelin (d18:1/18:0) | 794.602 | 130.607 | 1.460 | 0.038 | 0.663 |

**Table S5.** Pathway analysis of serum metabolomics in ISL and CON groups of fattening sheep.

| Pathway | Total | Hits | Raw P | Impact | Hits compounds |
| --- | --- | --- | --- | --- | --- |
| Arginine and proline metabolism | 44 | 3 | 0.005 | 0.149 | Citrulline cpd:C00327; Argininosuccinic acid cpd:C03406; L-Glutamic acid cpd:C00025 |
| Histidine metabolism | 14 | 2 | 0.006 | 0.130 | Urocanic acid cpd:C00785; Imidazoleacetic acid cpd:C02835 |
| Alanine, aspartate and glutamate metabolism | 23 | 2 | 0.016 | 0.293 | Argininosuccinic acid cpd:C03406; L-Glutamic acid cpd:C00025 |
| D-Glutamine and D-glutamate metabolism | 5 | 1 | 0.042 | 1 | L-Glutamic acid cpd:C00025 |
| Nitrogen metabolism | 9 | 1 | 0.075 | 0 | L-Glutamic acid cpd:C00025 |
| Valine, leucine and isoleucine biosynthesis | 11 | 1 | 0.091 | 0.333 | L-Isoleucine cpd:C00407 |
| Butanoate metabolism | 20 | 1 | 0.160 | 0 | L-Glutamic acid cpd:C00025 |
| Galactose metabolism | 26 | 1 | 0.203 | 0 | Galactitol cpd:C01697 |
| Glutathione metabolism | 26 | 1 | 0.203 | 0.055 | L-Glutamic acid cpd:C00025 |
| Glycine, serine and threonine metabolism | 32 | 1 | 0.245 | 0 | Betaine aldehyde cpd:C00576 |
| Valine, leucine and isoleucine degradation | 38 | 1 | 0.284 | 0 | L-Isoleucine cpd:C00407 |
| Biosynthesis of unsaturated fatty acids | 42 | 1 | 0.309 | 0 | Eicosapentaenoic acid cpd:C06428 |
| Drug metabolism - cytochrome P450 | 56 | 1 | 0.390 | 0 | Valproic acid cpd:C07185 |
| Aminoacyl-tRNA biosynthesis | 64 | 1 | 0.433 | 0 | L-Isoleucine cpd:C00407 |
| Pantothenate and CoA biosynthesis | 15 | 2 | 0.033 | 0.041 | Dihydrouracil cpd:C00429; Uracil cpd:C00106 |
| beta-Alanine metabolism | 17 | 2 | 0.041 | 0.130 | Dihydrouracil cpd:C00429; Uracil cpd:C00106 |
| Pyrimidine metabolism | 37 | 2 | 0.160 | 0.132 | Dihydrouracil cpd:C00429; Uracil cpd:C00106 |
| Caffeine metabolism | 12 | 1 | 0.210 | 0 | 7-Methylxanthine cpd:C16353 |
| Pentose phosphate pathway | 19 | 1 | 0.313 | 0 | D-Ribose cpd:C00121 |
| Citrate cycle (TCA cycle) | 20 | 1 | 0.326 | 0.072 | Pyruvic acid cpd:C00022 |
| Pyruvate metabolism | 22 | 1 | 0.352 | 0.188 | Pyruvic acid cpd:C00022 |
| Purine metabolism | 68 | 2 | 0.384 | 0.008 | Xanthosine cpd:C01762; Uric acid cpd:C00366 |
| Glycolysis or Gluconeogenesis | 26 | 1 | 0.402 | 0.099 | Pyruvic acid cpd:C00022 |
| Cysteine and methionine metabolism | 28 | 1 | 0.426 | 0.021 | Pyruvic acid cpd:C00022 |
| Tryptophan metabolism | 41 | 1 | 0.558 | 0.010 | Formylanthranilic acid cpd:C05653 |

**Table S6.** Significant differences in metabolites between ISL and CON groups in urine of fattening sheep.

| Name | MZ | R.T（min） | VIP | Pvalue | FC |
| --- | --- | --- | --- | --- | --- |
| **Negative** | | | | | |
| Pyrocatechol | 109.029 | 64.993 | 1.276 | 0.015 | 1.690 |
| 2-Hydroxy-3-methylbutyric acid | 117.055 | 224.983 | 1.402 | 0.048 | 0.511 |
| Isovaleric acid | 123.045 | 103.837 | 1.717 | 0.001 | 0.458 |
| Barbituric acid | 127.014 | 210.606 | 1.425 | 0.029 | 0.462 |
| Dihydroxyfumarate | 129.055 | 336.337 | 1.290 | 0.040 | 0.743 |
| Ethylmalonic acid | 131.035 | 311.162 | 1.442 | 0.046 | 0.504 |
| 5-Hydroxyhexanoic acid | 131.071 | 218.583 | 1.938 | 0.000 | 0.440 |
| Hydroxyisocaproic acid | 131.070 | 119.469 | 1.785 | 0.003 | 3.121 |
| 2-Methylbenzoic acid | 135.044 | 38.183 | 1.513 | 0.032 | 0.407 |
| Ethosuximide | 140.071 | 228.762 | 1.904 | 0.005 | 0.359 |
| Thymine | 142.066 | 188.278 | 1.292 | 0.036 | 0.472 |
| 2-Hydroxyphenylacetic acid | 151.039 | 222.274 | 1.672 | 0.044 | 0.393 |
| 3-Isopropylmalate | 157.049 | 131.265 | 1.713 | 0.009 | 0.404 |
| Pimelic acid | 159.066 | 337.602 | 1.195 | 0.042 | 0.776 |
| Betaine aldehyde | 160.097 | 68.970 | 1.687 | 0.020 | 0.394 |
| 4-Hydroxycoumarin | 161.023 | 48.481 | 1.727 | 0.001 | 0.421 |
| Acetyl-L-Cysteine | 162.022 | 227.324 | 1.557 | 0.044 | 0.249 |
| L-Tyrosine | 162.055 | 271.539 | 1.411 | 0.042 | 0.573 |
| 3-Hydroxymandelic acid | 168.044 | 188.583 | 1.253 | 0.049 | 0.464 |
| 4-Methoxycinnamic acid | 177.055 | 106.452 | 1.588 | 0.012 | 0.495 |
| L-homocysteic acid | 182.012 | 195.401 | 1.561 | 0.047 | 0.252 |
| 10-hydroxy capric acid | 187.132 | 74.179 | 1.406 | 0.020 | 0.487 |
| Pyridoxal (Vitamin B6) | 188.034 | 296.802 | 2.088 | 0.000 | 14.679 |
| sn-Glycerol 1-phosphate | 188.091 | 253.285 | 1.712 | 0.024 | 0.477 |
| Hexanoylglycine | 194.080 | 130.786 | 1.650 | 0.027 | 0.294 |
| Shikimate | 195.029 | 281.599 | 1.761 | 0.005 | 1.815 |
| Galactonic acid | 195.050 | 371.243 | 1.907 | 0.001 | 1.829 |
| Cyclohexylsulfamate | 195.077 | 27.596 | 1.540 | 0.034 | 0.329 |
| 3-Methylxanthine | 203.000 | 183.134 | 1.343 | 0.036 | 0.660 |
| 3-Prenyl-4-Hydroxyacetophenone | 203.106 | 125.756 | 1.260 | 0.028 | 0.521 |
| Caprylic acid | 203.127 | 205.179 | 1.258 | 0.049 | 0.587 |
| 5-Methoxyindoleacetate | 205.070 | 371.177 | 1.590 | 0.027 | 1.342 |
| D-Arabinono-1,4-lactone | 207.050 | 132.479 | 1.442 | 0.045 | 0.584 |
| Pentobarbital | 207.112 | 194.333 | 1.415 | 0.031 | 0.504 |
| 2-keto-D-Gluconic acid | 210.059 | 72.716 | 1.518 | 0.019 | 0.567 |
| Phosphocreatine | 211.038 | 277.954 | 1.405 | 0.144 | 0.601 |
| N1-Methyl-2-pyridone-5-carboxamide | 211.071 | 256.721 | 1.368 | 0.148 | 0.674 |
| L-Cysteinesulfinic acid | 212.022 | 249.468 | 1.342 | 0.145 | 0.428 |
| O-Succinyl-L-homoserine | 218.065 | 346.792 | 1.898 | 0.004 | 2.987 |
| 5-Hydroxy-L-lysine | 221.109 | 253.971 | 1.256 | 0.046 | 0.513 |
| N-Acetyl-L-tyrosine | 222.075 | 287.869 | 1.349 | 0.028 | 0.384 |
| L-Carnosine | 225.099 | 407.837 | 1.496 | 0.028 | 0.623 |
| 2'-Deoxyuridine | 227.069 | 127.288 | 2.017 | 0.001 | 0.181 |
| Butabarbital | 233.092 | 128.533 | 1.751 | 0.009 | 0.239 |
| d-Dethiobiotin | 235.107 | 124.939 | 1.851 | 0.008 | 0.270 |
| Dulcin | 239.109 | 116.893 | 1.871 | 0.012 | 0.211 |
| L-Cysteine | 241.027 | 114.348 | 2.037 | 0.000 | 17.166 |
| 4-Hydroxybenzaldehyde | 243.069 | 46.267 | 1.584 | 0.003 | 0.392 |
| 3-Phospho-D-glycerate | 245.011 | 33.075 | 1.686 | 0.037 | 0.521 |
| Pyridoxal 5'-phosphate | 247.026 | 34.455 | 1.584 | 0.029 | 0.417 |
| D-Neopterin | 252.076 | 29.174 | 1.703 | 0.001 | 1.807 |
| gamma-Glutamyl-L-methionine | 259.071 | 269.993 | 1.352 | 0.047 | 0.533 |
| Thymidine | 263.065 | 163.773 | 1.072 | 0.021 | 0.457 |
| Isoproturon | 265.152 | 134.308 | 1.891 | 0.014 | 0.207 |
| Inosine | 267.072 | 213.921 | 1.302 | 0.040 | 0.549 |
| Uridine | 281.017 | 296.587 | 1.665 | 0.003 | 0.356 |
| D-Ribose 5-phosphate | 289.036 | 37.498 | 1.390 | 0.030 | 0.636 |
| Anastrozole | 292.157 | 42.289 | 1.443 | 0.038 | 0.301 |
| Nitrofurantoin | 297.047 | 372.591 | 1.707 | 0.038 | 1.827 |
| Sulfaphenazole | 313.072 | 67.821 | 1.178 | 0.039 | 0.534 |
| beta-Octylglucoside | 313.162 | 268.436 | 1.726 | 0.003 | 0.480 |
| D-Mannitol 1-phosphate | 321.167 | 105.877 | 1.691 | 0.010 | 0.440 |
| Hexadecanedioic acid | 323.168 | 288.029 | 1.458 | 0.042 | 0.517 |
| Picrotoxinin | 329.047 | 331.237 | 1.898 | 0.001 | 2.240 |
| 15-keto-PGE1 | 333.203 | 131.996 | 1.484 | 0.030 | 0.406 |
| 3'-O-methylguanosine | 334.057 | 28.572 | 1.551 | 0.013 | 1.705 |
| Estrone 3-sulfate | 349.109 | 330.228 | 1.485 | 0.019 | 0.614 |
| D-Glucuronolactone | 351.065 | 304.658 | 1.263 | 0.047 | 0.568 |
| PGF3a | 351.213 | 105.115 | 1.464 | 0.049 | 0.507 |
| Phenolphthalein | 355.045 | 45.751 | 1.316 | 0.040 | 0.613 |
| 17alpha-Hydroxyprogesterone | 367.172 | 104.307 | 1.514 | 0.023 | 0.510 |
| 6k-PGF1alpha-d4 | 373.255 | 154.084 | 1.670 | 0.028 | 0.458 |
| Riboflavin | 375.126 | 203.673 | 1.270 | 0.046 | 0.526 |
| Flavin mononucleotide (FMN) | 455.095 | 47.191 | 1.480 | 0.006 | 0.486 |
| Adenylsuccinic acid | 462.058 | 36.029 | 1.946 | 0.000 | 2.174 |
| UDP-D-Galactose | 565.061 | 132.919 | 1.666 | 0.001 | 0.430 |
| Topiramate | 677.175 | 271.569 | 1.633 | 0.048 | 0.314 |
| **Postive** | | | | | |
| Methylguanidine | 74.071 | 256.314 | 1.985 | 0.014 | 2.562 |
| D-Ornithine | 115.085 | 199.950 | 1.575 | 0.014 | 0.659 |
| Tyramine | 120.079 | 217.181 | 1.371 | 0.046 | 0.568 |
| Salicylic acid | 121.028 | 220.666 | 1.501 | 0.021 | 0.593 |
| Taurine | 126.022 | 281.584 | 1.623 | 0.008 | 0.374 |
| 5-Methylcytosine | 126.065 | 188.997 | 1.708 | 0.038 | 0.442 |
| Quinolinate | 132.010 | 177.681 | 1.698 | 0.019 | 2.105 |
| 2-Ethoxyethanol | 132.101 | 327.828 | 1.620 | 0.012 | 0.498 |
| Perillyl alcohol | 135.115 | 64.578 | 1.273 | 0.034 | 0.581 |
| Dopamine | 136.074 | 207.310 | 1.519 | 0.010 | 0.544 |
| Ser-Ala | 141.065 | 95.001 | 1.471 | 0.019 | 0.608 |
| Acetyl-DL-Valine | 142.085 | 94.277 | 1.667 | 0.005 | 0.518 |
| 1-Aminocyclopropanecarboxylic acid | 143.081 | 321.818 | 1.616 | 0.024 | 0.562 |
| 2-Ketobutyric acid | 144.064 | 101.388 | 1.443 | 0.034 | 0.647 |
| Isovaleric acid | 144.100 | 290.159 | 1.506 | 0.019 | 1.494 |
| Coumarin | 147.042 | 201.549 | 1.556 | 0.031 | 0.494 |
| D-Ribose | 151.060 | 128.464 | 1.814 | 0.002 | 0.476 |
| Pyrocatechol | 152.069 | 266.988 | 1.615 | 0.038 | 1.608 |
| Xylitol | 153.072 | 283.425 | 1.994 | 0.020 | 2.263 |
| Phenol | 155.069 | 106.249 | 1.877 | 0.006 | 0.227 |
| Arecoline | 156.100 | 43.190 | 1.517 | 0.011 | 0.561 |
| Malonic acid | 165.037 | 127.101 | 1.556 | 0.025 | 0.525 |
| 3-Methoxytyramine | 168.106 | 117.170 | 1.864 | 0.001 | 0.291 |
| Hydroxyproline | 170.020 | 144.790 | 1.840 | 0.002 | 2.289 |
| (S)-(-)-Citronellic acid | 171.137 | 217.366 | 1.263 | 0.038 | 0.597 |
| L-Kynurenine | 173.069 | 209.563 | 2.120 | 0.011 | 6.750 |
| Succinate | 179.051 | 33.044 | 1.375 | 0.017 | 0.517 |
| Valproic acid | 186.147 | 196.032 | 1.614 | 0.042 | 0.433 |
| Jasmonic acid | 193.120 | 184.510 | 1.432 | 0.015 | 0.556 |
| N-Phenylacetamide | 196.095 | 80.847 | 1.722 | 0.009 | 0.483 |
| 3-Guanidinopropanoate | 198.021 | 40.265 | 1.398 | 0.033 | 0.513 |
| Phosphoglycolic acid | 198.060 | 152.730 | 2.205 | 0.002 | 6.012 |
| N-Acetyl-L-Histidine | 198.085 | 292.824 | 1.978 | 0.019 | 1.949 |
| Coniferol | 198.110 | 157.218 | 1.473 | 0.017 | 0.551 |
| L-Tryptophan | 205.095 | 179.176 | 1.371 | 0.029 | 0.475 |
| 3,4-Dimethoxycinnamic acid | 208.078 | 143.778 | 1.297 | 0.012 | 0.487 |
| D-Xylose | 211.078 | 210.453 | 1.492 | 0.020 | 0.476 |
| Suberylglycine | 214.106 | 326.623 | 1.410 | 0.036 | 0.446 |
| N-Acetylserotonin | 219.111 | 50.050 | 1.813 | 0.003 | 2.984 |
| Carbaryl | 219.111 | 71.534 | 2.133 | 0.003 | 6.320 |
| 3-Indoleacetonitrile | 223.019 | 231.912 | 1.544 | 0.031 | 0.653 |
| 5-Hydroxy-L-lysine | 226.116 | 256.021 | 1.519 | 0.046 | 1.881 |
| Terbutaline | 226.142 | 126.142 | 1.514 | 0.039 | 0.413 |
| Ethionamide | 227.080 | 38.214 | 2.140 | 0.007 | 4.355 |
| alpha-Guanidinoglutaric Acid | 228.033 | 29.948 | 1.750 | 0.004 | 0.521 |
| Val-Asp | 232.106 | 67.446 | 1.946 | 0.000 | 0.277 |
| Swainsonine | 234.130 | 194.725 | 1.559 | 0.032 | 0.562 |
| Equol | 243.100 | 197.768 | 1.346 | 0.032 | 0.612 |
| Lamivudine | 247.090 | 165.967 | 1.245 | 0.048 | 0.620 |
| Methylone | 249.123 | 114.111 | 1.610 | 0.045 | 0.342 |
| Normetanephrine | 250.044 | 48.751 | 1.979 | 0.041 | 0.224 |
| Acetyl Tyrosine Ethyl Ester | 251.113 | 89.847 | 1.720 | 0.039 | 0.328 |
| Caffeine | 255.106 | 264.663 | 1.638 | 0.013 | 0.525 |
| Ile-Thr | 255.133 | 485.522 | 1.788 | 0.019 | 0.305 |
| 3-Hydroxyflavone | 256.103 | 233.994 | 1.645 | 0.019 | 0.526 |
| L-Pyroglutamic acid | 259.095 | 226.565 | 1.573 | 0.010 | 0.483 |
| Ile-Tyr | 259.142 | 194.579 | 1.286 | 0.049 | 0.299 |
| Arg-Cys | 260.112 | 165.375 | 1.724 | 0.003 | 0.320 |
| Metyrapone | 265.072 | 33.451 | 0.947 | 0.026 | 6.131 |
| Gly-Glu | 265.106 | 228.713 | 1.631 | 0.010 | 0.666 |
| Guanosine | 266.087 | 135.028 | 1.284 | 0.032 | 1.645 |
| Apigenin | 271.058 | 43.113 | 1.335 | 0.048 | 0.509 |
| D-Galactarate | 271.068 | 289.544 | 1.597 | 0.037 | 1.786 |
| Ergothioneine | 272.132 | 264.663 | 1.490 | 0.028 | 0.583 |
| Dihydro-4,4-dimethyl-2,3-furandione | 274.127 | 342.807 | 1.869 | 0.039 | 2.190 |
| Palmitic acid | 274.272 | 63.210 | 1.387 | 0.047 | 0.429 |
| Val-Leu | 275.130 | 342.832 | 1.853 | 0.029 | 1.966 |
| Glycitein | 285.074 | 33.086 | 1.2888 | 0.049 | 1.878 |
| Gemcitabine | 286.056 | 176.096 | 1.415 | 0.030 | 0.678 |
| Nortriptyline | 286.163 | 181.948 | 1.468 | 0.014 | 0.472 |
| Dapsone | 287.031 | 33.749 | 2.173 | 0.003 | 9.822 |
| Tolbutamide | 288.132 | 50.003 | 1.519 | 0.047 | 1.904 |
| Sotalol | 290.159 | 371.876 | 1.537 | 0.018 | 1.739 |
| Phenelzine | 295.188 | 65.776 | 1.445 | 0.022 | 0.633 |
| Triphenyltetrazolium | 299.134 | 260.836 | 1.489 | 0.041 | 0.611 |
| Acetomenaphthone | 300.119 | 28.642 | 1.289 | 0.018 | 0.567 |
| Ile-Lys | 301.217 | 199.704 | 1.521 | 0.021 | 0.478 |
| N-Acetylaspartylglutamate (NAAG) | 305.096 | 404.854 | 1.438 | 0.044 | 1.707 |
| Promethazine | 307.126 | 262.689 | 1.966 | 0.013 | 2.473 |
| Trp-Cys | 308.108 | 213.680 | 1.383 | 0.021 | 0.552 |
| Tyr-Phe | 311.147 | 97.723 | 1.461 | 0.046 | 0.641 |
| N2,N2-Dimethylguanosine | 312.129 | 184.201 | 1.464 | 0.050 | 0.485 |
| Tyr-Ala | 313.133 | 184.106 | 1.534 | 0.037 | 0.506 |
| Ipriflavone | 319.067 | 25.551 | 2.135 | 0.002 | 42.411 |
| 2-Oxoadipic acid | 321.086 | 46.427 | 1.276 | 0.022 | 2.421 |
| Fluoxetine | 332.125 | 233.997 | 1.815 | 0.008 | 0.500 |
| Formononetin | 335.031 | 35.247 | 2.245 | 0.002 | 136.580 |
| gamma-L-Glutamyl-L-phenylalanine | 336.163 | 140.228 | 1.399 | 0.031 | 0.497 |
| beta-Octylglucoside | 337.165 | 396.927 | 1.547 | 0.040 | 0.466 |
| Prostaglandin B1 | 337.235 | 80.437 | 1.530 | 0.002 | 0.454 |
| 2-C-Methyl-D-erythritol 2,4-cyclodiphosphate | 339.023 | 38.528 | 1.893 | 0.006 | 0.228 |
| Demethoxycurcumin | 339.131 | 235.708 | 1.419 | 0.044 | 0.493 |
| Arg-Thr | 352.076 | 404.833 | 1.730 | 0.027 | 0.504 |
| Tyr-Glu | 352.145 | 296.356 | 2.008 | 0.005 | 1.847 |
| Arg-Ile | 354.141 | 49.678 | 1.852 | 0.009 | 3.134 |
| Tetrahydrocortisone | 365.229 | 96.818 | 1.327 | 0.028 | 0.538 |
| Chlorogenic acid | 372.127 | 272.789 | 2.084 | 0.009 | 2.219 |
| Mefloquine | 378.113 | 415.061 | 1.313 | 0.041 | 0.637 |
| Norfloxacin | 380.171 | 89.033 | 2.079 | 0.017 | 0.155 |
| Olanzapine | 389.061 | 103.005 | 1.842 | 0.028 | 2.607 |
| Midazolam | 389.092 | 43.451 | 1.770 | 0.015 | 0.553 |
| Decanoyl-L-carnitine | 393.166 | 345.087 | 1.659 | 0.044 | 1.834 |
| Trp-Gln | 399.105 | 226.644 | 1.660 | 0.019 | 0.483 |
| Trimethobenzamide | 406.226 | 399.227 | 1.439 | 0.046 | 0.531 |
| Aloin A | 418.133 | 48.629 | 1.904 | 0.022 | 2.566 |
| Pantoprazole | 425.113 | 234.945 | 1.636 | 0.032 | 0.557 |
| 5,10-methylene-THF | 440.178 | 192.347 | 1.701 | 0.010 | 0.350 |
| Cytidine 5'-diphosphocholine (CDP-choline) | 489.112 | 199.244 | 1.285 | 0.045 | 0.589 |
| 1-Palmitoyl-sn-glycero-3-phosphocholine | 496.335 | 181.366 | 1.397 | 0.018 | 0.525 |
| Taurodeoxycholic acid | 538.258 | 198.567 | 1.387 | 0.034 | 0.638 |

**Table S7.** Pathway analysis of urine metabolomics in ISL and CON groups of fattening sheep.

| Pathway | Total | Hits | Raw P | Impact | Hits compunds |
| --- | --- | --- | --- | --- | --- |
| Tryptophan metabolism | 41 | 4 | 0.060 | 0.259 | L-Tryptophan cpd:C00078; N-Acetylserotonin cpd:C00978; Oxoadipic acid cpd:C00322; L-Kynurenine cpd:C00328 |
| Pentose and glucuronate interconversions | 15 | 2 | 0.102 | 0.083 | D-Xylitol cpd:C00379; D-Xylose cpd:C00181 |
| Glyoxylate and dicarboxylate metabolism | 16 | 2 | 0.114 | 0 | Phosphoglycolic acid cpd:C00988; 5,10-Methylene-THF cpd:C00143 |
| D-Arginine and D-ornithine metabolism | 4 | 1 | 0.139 | 0 | D-Ornithine cpd:C00515 |
| Tyrosine metabolism | 42 | 3 | 0.197 | 0.223 | Dopamine cpd:C03758; 3-Methoxytyramine cpd:C05587; Tyramine cpd:C00483 |
| Taurine and hypotaurine metabolism | 7 | 1 | 0.231 | 0.750 | Taurine cpd:C00245 |
| One carbon pool by folate | 8 | 1 | 0.259 | 0.039 | 5,10-Methylene-THF cpd:C00143 |
| Methane metabolism | 9 | 1 | 0.286 | 0.400 | 5,10-Methylene-THF cpd:C00143 |
| Caffeine metabolism | 12 | 1 | 0.362 | 0 | Caffeine cpd:C07481 |
| Nicotinate and nicotinamide metabolism | 13 | 1 | 0.386 | 0 | Quinolinic acid cpd:C03722 |
| Pentose phosphate pathway | 19 | 1 | 0.511 | 0 | D-Ribose cpd:C00121 |
| Propanoate metabolism | 20 | 1 | 0.529 | 0 | Succinic acid cpd:C00042 |
| Butanoate metabolism | 20 | 1 | 0.529 | 0 | Succinic acid cpd:C00042 |
| Citrate cycle (TCA cycle) | 20 | 1 | 0.529 | 0.026 | Succinic acid cpd:C00042 |
| Lysine degradation | 20 | 1 | 0.529 | 0.090 | Oxoadipic acid cpd:C00322 |
| Alanine, aspartate and glutamate metabolism | 23 | 1 | 0.579 | 0 | Succinic acid cpd:C00042 |
| Glutathione metabolism | 26 | 1 | 0.625 | 0.014 | Pyroglutamic acid cpd:C01879 |
| Fatty acid elongation in mitochondria | 27 | 1 | 0.639 | 0 | Palmitic acid cpd:C00249 |
| Glycerophospholipid metabolism | 29 | 1 | 0.665 | 0.067 | Citicoline cpd:C00307 |
| Glycine, serine and threonine metabolism | 32 | 1 | 0.702 | 0.046 | 5,10-Methylene-THF cpd:C00143 |
| Fatty acid biosynthesis | 38 | 1 | 0.763 | 0 | Palmitic acid cpd:C00249 |
| Fatty acid metabolis | 39 | 1 | 0.772 | 0 | Palmitic acid cpd:C00249 |
| Biosynthesis of unsaturated fatty acids | 42 | 1 | 0.797 | 0 | Palmitic acid cpd:C00249 |
| Arginine and proline metabolism | 44 | 1 | 0.812 | 0.041 | Hydroxyproline cpd:C01157 |
| Primary bile acid biosynthesis | 46 | 1 | 0.826 | 0.030 | Taurine cpd:C00245 |
| Drug metabolism - cytochrome P450 | 56 | 1 | 0.882 | 0 | Valproic acid cpd:C07185 |
| Aminoacyl-tRNA biosynthesis | 64 | 1 | 0.913 | 0 | L-Tryptophan cpd:C00078 |
| Purine metabolism | 68 | 1 | 0.926 | 0.003 | Guanosine cpd:C00387 |
| Pyrimidine metabolism | 37 | 4 | 0.021 | 0.079 | Uridine cpd:C00299; Deoxyuridine cpd:C00526; Thymidine cpd:C00214; Thymine cpd:C00178 |
| Phenylalanine metabolism | 9 | 2 | 0.027 | 0 | Ortho-Hydroxyphenylacetic acid cpd:C05852; L-Tyrosine cpd:C00082 |
| Vitamin B6 metabolism | 9 | 2 | 0.027 | 0.745 | Pyridoxal 5'-phosphate cpd:C00018; Pyridoxal cpd:C00250 |
| Riboflavin metabolism | 11 | 2 | 0.039 | 0.333 | Flavin Mononucleotide cpd:C00061; Riboflavin cpd:C00255 |
| Ubiquinone and other terpenoid-quinone biosynthesis | 3 | 1 | 0.086 | 0 | L-Tyrosine cpd:C00082 |
| Phenylalanine, tyrosine and tryptophan biosynthesis | 4 | 1 | 0.113 | 0.500 | L-Tyrosine cpd:C00082 |
| Thiamine metabolism | 7 | 1 | 0.189 | 0 | L-Tyrosine cpd:C00082 |
| Cysteine and methionine metabolism | 28 | 2 | 0.199 | 0.151 | L-Cysteine cpd:C00097; 3-Sulfinoalanine cpd:C00606 |
| Ascorbate and aldarate metabolism | 9 | 1 | 0.237 | 0 | D-Glucurono-6,3-lactone cpd:C02670 |
| Histidine metabolism | 14 | 1 | 0.344 | 0 | Carnosine cpd:C00386 |
| Pantothenate and CoA biosynthesis | 15 | 1 | 0.363 | 0 | L-Cysteine cpd:C00097 |
| Galactose metabolism | 26 | 1 | 0.544 | 0.005 | Uridine diphosphategalactose cpd:C00052 |
| Steroid hormone biosynthesis | 67 | 2 | 0.598 | 0.050 | 17-Hydroxyprogesterone cpd:C01176; Estrone sulfate cpd:C02538 |
| Amino sugar and nucleotide sugar metabolism | 37 | 1 | 0.674 | 0 | Uridine diphosphategalactose cpd:C00052 |

**Table S8.** Analysis of significant differential expression of circRNA between ISL group and CON group.

| circRNA id^1^ | MeanRPKM^2^  (ISL) | MeanRPKM (CON) | log2FoldChange^3^ | Pvalue^4^ |
| --- | --- | --- | --- | --- |
| **Up** | | | | |
| circRNA01712 | 740.106 | 175.609 | 2.075 | 0.008 |
| circRNA04645 | 83.760 | 0.000 | 19.676 | 0.026 |
| circRNA04624 | 113.468 | 0.000 | 20.114 | 0.036 |
| circRNA01756 | 218.240 | 0.000 | 21.057 | 0.029 |
| circRNA00857 | 338.174 | 132.164 | 1.355 | 0.020 |
| circRNA04615 | 1037.865 | 0.000 | 23.307 | 0.015 |
| circRNA04559 | 300.495 | 0.000 | 21.519 | 0.049 |
| circRNA01821 | 1438.985 | 256.319 | 2.489 | 0.049 |
| circRNA04534 | 47.761 | 0.000 | 18.865 | 0.025 |
| circRNA01016 | 229.927 | 0.000 | 21.133 | 0.038 |
| **Down** | | | | |
| circRNA04308 | 0.000 | 2166.905 | -24.369 | 0.008 |

^1^ circRNA ID: Transcript number.

^2^ MeanTPM: Expression level of grouping.

^3^ log2FoldChange: log2 value of difference multiple.

^4^ Pvalue: Statistical significance test indicators.

**Table S9.** 30 significantly enriched differential circRNAs in the ISL diet group.

| GO.ID^1^ | Term^2^ | Ontology^3^ | Significant^4^ | Annotated^5^ | Pvalue |
| --- | --- | --- | --- | --- | --- |
| GO:2001252 | positive regulation of chromosome organization | biological process | 2/9 | 133/14620 | 0.003 |
| GO:1990164 | histone H2A phosphorylation | biological process | 1/9 | 3/14620 | 0.002 |
| GO:1902287 | semaphorin-plexin signaling pathway involved in axon guidance | biological process | 1/9 | 5/14620 | 0.003 |
| GO:1902285 | semaphorin-plexin signaling pathway involved in neuron projection guidance | biological process | 1/9 | 6/14620 | 0.004 |
| GO:1901166 | neural crest cell migration involved in autonomic nervous system development | biological process | 1/9 | 5/14620 | 0.003 |
| GO:0097491 | sympathetic neuron projection guidance | biological process | 1/9 | 4/14620 | 0.002 |
| GO:0097490 | sympathetic neuron projection extension | biological process | 1/9 | 4/14620 | 0.002 |
| GO:0071840 | cellular component organization or biogenesis | biological process | 8/9 | 4080/14620 | 0.000 |
| GO:0061549 | sympathetic ganglion development | biological process | 1/9 | 6/14620 | 0.004 |
| GO:0051128 | regulation of cellular component organization | biological process | 7/9 | 1616/14620 | 0.000 |
| GO:0046832 | negative regulation of RNA export from nucleus | biological process | 1/9 | 2/14620 | 0.001 |
| GO:0043990 | histone H2A-S1 phosphorylation | biological process | 1/9 | 1/14620 | 0.001 |
| GO:0043988 | histone H3-S10 phosphorylation | biological process | 1/9 | 3/14620 | 0.002 |
| GO:0043987 | histone H3-S28 phosphorylation | biological process | 1/9 | 3/14620 | 0.002 |
| GO:0036486 | ventral trunk neural crest cell migration | biological process | 1/9 | 4/14620 | 0.002 |
| GO:0036484 | trunk neural crest cell migration | biological process | 1/9 | 4/14620 | 0.002 |
| GO:0035404 | histone-serine phosphorylation | biological process | 1/9 | 7/14620 | 0.004 |
| GO:0035290 | trunk segmentation | biological process | 1/9 | 4/14620 | 0.002 |
| GO:0033129 | positive regulation of histone phosphorylation | biological process | 1/9 | 5/14620 | 0.003 |
| GO:0032989 | cellular component morphogenesis | biological process | 4/9 | 698/14620 | 0.001 |
| GO:0032240 | negative regulation of nucleobase-containing compound transport | biological process | 1/9 | 2/14620 | 0.001 |
| GO:0022604 | regulation of cell morphogenesis | biological process | 4/9 | 276/14620 | 0.000 |
| GO:0022603 | regulation of anatomical structure morphogenesis | biological process | 4/9 | 647/14620 | 0.000 |
| GO:0021785 | branchiomotor neuron axon guidance | biological process | 1/9 | 6/14620 | 0.004 |
| GO:0021637 | trigeminal nerve structural organization | biological process | 1/9 | 5/14620 | 0.003 |
| GO:0021636 | trigeminal nerve morphogenesis | biological process | 1/9 | 5/14620 | 0.003 |
| GO:0021559 | trigeminal nerve development | biological process | 1/9 | 7/14620 | 0.004 |
| GO:0016043 | cellular component organization | biological process | 8/9 | 3983/14620 | 0.000 |
| GO:0006996 | organelle organization | biological process | 6/9 | 2462/14620 | 0.001 |
| GO:0000902 | cell morphogenesis | biological process | 4/9 | 633/14620 | 0.000 |

^1^ GO.ID：Number and name of GO.

^2^ Term：Description information of GO function.

^3^ Ontology：Category of GO（cellular component；biological process；molecular function）.

^4^ Significant：Number of differentially expressed circRNA host genes annotated to the GO/Number of differentially expressed circRNA host genes annotated to the GO database.

^5^ Annotated：Number of genes annotated to the GO/Number of genes annotated to the GO data-base.

**Table S10.** Significant differential expression analysis of transcripts (lncRNA, mRNA) between ISL group and CON group.

| **Transcript id**^1^ | **MeanTPM (ISL)** | **MeanTPM (CON)** | **log2FoldChange** | **Pvalue** |
| --- | --- | --- | --- | --- |
| **Up** | | | | |
| MSTRG.81411.1 | 9.743 | 4.800 | 1.021 | 0.000 |
| MSTRG.60584.10 | 10.557 | 5.047 | 1.065 | 0.000 |
| MSTRG.68377.1 | 20.090 | 8.137 | 1.304 | 0.000 |
| MSTRG.31159.1 | 5.140 | 1.467 | 1.809 | 0.000 |
| MSTRG.23357.8 | 15.080 | 0.703 | 4.422 | 0.000 |
| MSTRG.91313.7 | 10.163 | 3.987 | 1.350 | 0.000 |
| MSTRG.20120.1 | 7.470 | 2.867 | 1.382 | 0.000 |
| MSTRG.69743.4 | 8.973 | 4.407 | 1.026 | 0.000 |
| MSTRG.11909.10 | 5.277 | 2.267 | 1.219 | 0.000 |
| MSTRG.82103.2 | 7.930 | 3.560 | 1.155 | 0.000 |
| MSTRG.66952.9 | 9.510 | 4.717 | 1.012 | 0.000 |
| MSTRG.30934.2 | 7.007 | 2.823 | 1.311 | 0.000 |
| MSTRG.20111.1 | 5.863 | 2.343 | 1.323 | 0.000 |
| MSTRG.8613.39 | 16.483 | 6.370 | 1.372 | 0.000 |
| MSTRG.85322.2 | 5.117 | 2.383 | 1.102 | 0.000 |
| MSTRG.68377.28 | 30.157 | 12.193 | 1.306 | 0.000 |
| MSTRG.38991.16 | 8.130 | 3.967 | 1.035 | 0.000 |
| MSTRG.81402.16 | 14.307 | 4.413 | 1.697 | 0.000 |
| MSTRG.96530.1 | 7.987 | 3.810 | 1.068 | 0.000 |
| MSTRG.86726.2 | 5.337 | 2.380 | 1.165 | 0.000 |
| MSTRG.72097.4 | 15.603 | 7.190 | 1.118 | 0.000 |
| MSTRG.2539.1 | 6.537 | 2.223 | 1.556 | 0.000 |
| MSTRG.12330.1 | 6.980 | 3.113 | 1.165 | 0.000 |
| MSTRG.4287.1 | 519.227 | 32.703 | 3.989 | 0.000 |
| MSTRG.4953.13 | 13.883 | 6.737 | 1.043 | 0.000 |
| MSTRG.40606.2 | 8.193 | 2.687 | 1.609 | 0.000 |
| MSTRG.81098.1 | 5.150 | 2.353 | 1.130 | 0.000 |
| MSTRG.17012.10 | 6.847 | 2.877 | 1.251 | 0.000 |
| MSTRG.12666.1 | 5.177 | 2.510 | 1.044 | 0.000 |
| MSTRG.76461.1 | 38.737 | 15.763 | 1.297 | 0.000 |
| MSTRG.20054.2 | 7.597 | 3.560 | 1.093 | 0.000 |
| MSTRG.54640.23 | 5.897 | 2.637 | 1.161 | 0.000 |
| MSTRG.76459.6 | 19.577 | 9.263 | 1.080 | 0.000 |
| MSTRG.33431.1 | 9.423 | 4.690 | 1.007 | 0.000 |
| MSTRG.89065.3 | 7.243 | 2.800 | 1.371 | 0.000 |
| MSTRG.46968.1 | 6.507 | 3.140 | 1.051 | 0.000 |
| MSTRG.82110.2 | 15.347 | 6.537 | 1.231 | 0.000 |
| MSTRG.20040.1 | 6.947 | 2.347 | 1.566 | 0.000 |
| MSTRG.89063.10 | 8.037 | 3.193 | 1.332 | 0.000 |
| MSTRG.30929.1 | 6.933 | 2.747 | 1.336 | 0.000 |
| MSTRG.1627.1 | 5.813 | 2.133 | 1.446 | 0.000 |
| MSTRG.16260.8 | 6.363 | 1.033 | 2.622 | 0.000 |
| MSTRG.30948.1 | 5.727 | 1.913 | 1.582 | 0.000 |
| MSTRG.56637.6 | 10.507 | 3.090 | 1.766 | 0.000 |
| MSTRG.60669.1 | 8.710 | 3.100 | 1.490 | 0.000 |
| MSTRG.75886.1 | 13.927 | 2.503 | 2.476 | 0.000 |
| MSTRG.31777.1 | 5.227 | 1.327 | 1.978 | 0.000 |
| MSTRG.20121.1 | 8.977 | 3.347 | 1.423 | 0.000 |
| MSTRG.76454.1 | 19.480 | 8.863 | 1.136 | 0.000 |
| MSTRG.64481.2 | 6.560 | 3.263 | 1.007 | 0.000 |
| MSTRG.63450.4 | 10.627 | 4.443 | 1.258 | 0.000 |
| MSTRG.28018.1 | 7.110 | 2.817 | 1.336 | 0.000 |
| MSTRG.85262.22 | 13.197 | 6.307 | 1.065 | 0.000 |
| MSTRG.7533.2 | 10.777 | 3.500 | 1.622 | 0.000 |
| MSTRG.69298.8 | 8.577 | 3.603 | 1.251 | 0.000 |
| MSTRG.62082.2 | 5.300 | 0.727 | 2.867 | 0.000 |
| MSTRG.50733.1 | 6.627 | 2.830 | 1.227 | 0.000 |
| MSTRG.79331.5 | 15.447 | 7.633 | 1.017 | 0.000 |
| MSTRG.71628.4 | 5.720 | 1.937 | 1.562 | 0.000 |
| MSTRG.41574.1 | 7.013 | 3.377 | 1.055 | 0.000 |
| MSTRG.26282.1 | 5.033 | 2.040 | 1.303 | 0.000 |
| MSTRG.43846.1 | 5.423 | 2.273 | 1.254 | 0.000 |
| MSTRG.4183.5 | 10.963 | 5.347 | 1.036 | 0.000 |
| ENSOART00000029046 | 268.067 | 101.120 | 1.407 | 0.000 |
| ENSOART00000020358 | 5.807 | 0.073 | 6.307 | 0.000 |
| ENSOART00000003995 | 33.653 | 12.853 | 1.389 | 0.000 |
| ENSOART00000005283 | 15.760 | 5.123 | 1.621 | 0.000 |
| ENSOART00000018298 | 52.103 | 24.320 | 1.099 | 0.000 |
| ENSOART00000021732 | 26.910 | 13.157 | 1.032 | 0.000 |
| ENSOART00000005653 | 5.040 | 1.303 | 1.951 | 0.000 |
| ENSOART00000010885 | 42.677 | 9.277 | 2.202 | 0.000 |
| ENSOART00000003721 | 3219.370 | 4.967 | 9.340 | 0.000 |
| ENSOART00000017336 | 19.200 | 2.220 | 3.112 | 0.000 |
| ENSOART00000020958 | 66.470 | 25.030 | 1.409 | 0.000 |
| ENSOART00000009894 | 766.000 | 16.040 | 5.578 | 0.000 |
| ENSOART00000008854 | 20.677 | 1.057 | 4.290 | 0.000 |
| ENSOART00000008074 | 5.470 | 2.537 | 1.109 | 0.000 |
| ENSOART00000003774 | 27.173 | 13.437 | 1.016 | 0.000 |
| ENSOART00000003636 | 574.937 | 1.077 | 9.061 | 0.000 |
| ENSOART00000014350 | 32.157 | 10.253 | 1.649 | 0.000 |
| ENSOART00000014259 | 11.920 | 3.507 | 1.765 | 0.000 |
| ENSOART00000019879 | 15.223 | 5.727 | 1.411 | 0.000 |
| ENSOART00000020939 | 46.140 | 18.263 | 1.337 | 0.000 |
| ENSOART00000000328 | 17.453 | 7.287 | 1.260 | 0.000 |
| ENSOART00000010169 | 134.730 | 30.703 | 2.134 | 0.000 |
| ENSOART00000015713 | 11.517 | 3.740 | 1.623 | 0.000 |
| ENSOART00000007510 | 62.423 | 25.897 | 1.269 | 0.000 |
| ENSOART00000004098 | 20.050 | 9.560 | 1.069 | 0.000 |
| ENSOART00000017275 | 377.487 | 136.857 | 1.464 | 0.000 |
| ENSOART00000002985 | 5.153 | 0.833 | 2.629 | 0.000 |
| ENSOART00000011004 | 18.427 | 7.317 | 1.333 | 0.000 |
| ENSOART00000019945 | 10.827 | 4.833 | 1.163 | 0.000 |
| ENSOART00000006176 | 12.583 | 6.287 | 1.001 | 0.000 |
| ENSOART00000003386 | 25.327 | 3.033 | 3.062 | 0.000 |
| **Down** | | | | |
| MSTRG.13969.6 | 0.000 | 5.890 | -15.846 | 0.000 |
| MSTRG.23970.1 | 1.160 | 9.597 | -3.048 | 0.000 |
| MSTRG.87688.1 | 0.003 | 6.573 | -10.945 | 0.000 |
| MSTRG.35424.1 | 0.157 | 5.450 | -5.120 | 0.000 |
| MSTRG.25906.2 | 76.640 | 164.840 | -1.105 | 0.000 |
| MSTRG.25946.1 | 3.450 | 9.417 | -1.449 | 0.000 |
| MSTRG.25405.2 | 2.397 | 7.873 | -1.716 | 0.000 |
| MSTRG.77996.2 | 6.507 | 16.420 | -1.335 | 0.000 |
| MSTRG.90666.7 | 3.473 | 9.827 | -1.500 | 0.000 |
| MSTRG.53194.10 | 2.883 | 26.667 | -3.209 | 0.000 |
| MSTRG.16260.13 | 0.013 | 5.653 | -8.728 | 0.000 |
| MSTRG.36400.1 | 1.070 | 15.493 | -3.856 | 0.000 |
| MSTRG.90841.1 | 2.027 | 6.277 | -1.631 | 0.000 |
| ENSOART00000006058 | 15.023 | 35.453 | -1.239 | 0.000 |
| ENSOART00000022234 | 10.800 | 24.753 | -1.197 | 0.000 |
| ENSOART00000020266 | 11.003 | 39.090 | -1.829 | 0.000 |
| ENSOART00000000362 | 6.010 | 14.393 | -1.260 | 0.000 |
| ENSOART00000005140 | 56.337 | 145.447 | -1.368 | 0.000 |
| ENSOART00000000477 | 227.137 | 945.687 | -2.058 | 0.000 |
| ENSOART00000011961 | 1.963 | 8.327 | -2.084 | 0.000 |
| ENSOART00000000735 | 0.000 | 7.543 | -16.203 | 0.000 |
| ENSOART00000021147 | 76.573 | 245.910 | -1.683 | 0.000 |
| ENSOART00000020009 | 11.800 | 26.227 | -1.152 | 0.000 |
| ENSOART00000022084 | 50.680 | 105.500 | -1.058 | 0.000 |
| ENSOART00000006453 | 3.370 | 7.003 | -1.055 | 0.000 |
| ENSOART00000016107 | 10.503 | 27.070 | -1.366 | 0.000 |
| ENSOART00000000483 | 1.713 | 5.513 | -1.686 | 0.000 |
| ENSOART00000017032 | 5.480 | 12.040 | -1.136 | 0.000 |
| ENSOART00000011132 | 9.537 | 43.113 | -2.177 | 0.000 |
| ENSOART00000012655 | 22.380 | 55.780 | -1.318 | 0.000 |
| ENSOART00000012418 | 0.577 | 8.573 | -3.894 | 0.000 |
| ENSOART00000012416 | 41.840 | 99.700 | -1.253 | 0.000 |
| ENSOART00000001738 | 33.393 | 105.070 | -1.654 | 0.000 |
| ENSOART00000015779 | 0.040 | 47.013 | -10.199 | 0.000 |
| ENSOART00000009914 | 41.657 | 94.600 | -1.183 | 0.000 |
| ENSOART00000008853 | 0.000 | 21.480 | -17.713 | 0.000 |
| ENSOART00000000795 | 3.463 | 10.630 | -1.618 | 0.000 |
| ENSOART00000002554 | 2.140 | 11.177 | -2.385 | 0.000 |
| ENSOART00000017040 | 4.373 | 30.747 | -2.814 | 0.000 |
| ENSOART00000015990 | 1.257 | 7.317 | -2.542 | 0.000 |
| ENSOART00000015451 | 3.147 | 7.693 | -1.290 | 0.000 |
| ENSOART00000018756 | 1.097 | 52.057 | -5.569 | 0.000 |
| ENSOART00000005586 | 6.130 | 12.700 | -1.051 | 0.000 |
| ENSOART00000011670 | 3.103 | 7.417 | -1.257 | 0.000 |
| ENSOART00000009657 | 5.767 | 18.700 | -1.697 | 0.000 |
| ENSOART00000007669 | 3.153 | 7.750 | -1.297 | 0.000 |
| ENSOART00000013601 | 3.230 | 8.560 | -1.406 | 0.000 |
| ENSOART00000015509 | 2.983 | 11.287 | -1.920 | 0.000 |
| ENSOART00000018685 | 0.697 | 8.737 | -3.649 | 0.000 |
| ENSOART00000022712 | 185.247 | 422.400 | -1.189 | 0.000 |
| ENSOART00000005620 | 44.923 | 277.410 | -2.626 | 0.000 |
| ENSOART00000012053 | 31.633 | 110.267 | -1.801 | 0.000 |
| ENSOART00000013725 | 1.730 | 6.417 | -1.891 | 0.000 |
| ENSOART00000001229 | 4.727 | 9.853 | -1.060 | 0.000 |
| ENSOART00000019133 | 66.097 | 198.337 | -1.585 | 0.000 |
| ENSOART00000004142 | 4.827 | 15.030 | -1.639 | 0.000 |
| ENSOART00000015928 | 10.510 | 31.717 | -1.593 | 0.000 |
| ENSOART00000014034 | 1.627 | 6.587 | -2.018 | 0.000 |
| ENSOART00000012456 | 55.800 | 120.683 | -1.113 | 0.000 |
| ENSOART00000010846 | 24.013 | 54.607 | -1.185 | 0.000 |
| ENSOART00000006465 | 41.337 | 90.807 | -1.135 | 0.000 |
| ENSOART00000015276 | 41.480 | 98.020 | -1.241 | 0.000 |
| ENSOART00000006792 | 0.007 | 6.563 | -9.943 | 0.000 |
| ENSOART00000008375 | 3.130 | 9.403 | -1.587 | 0.000 |
| ENSOART00000021938 | 22.137 | 52.033 | -1.233 | 0.000 |
| ENSOART00000000071 | 8.230 | 27.967 | -1.765 | 0.000 |

^1^ Transcript ID：Transcript number.

**Table S11.** Gene functions corresponding to 30 significantly enriched differential transcripts in the ISL group.

| GO.ID | Term | Ontology | Significant | Annotated | Pvalue |
| --- | --- | --- | --- | --- | --- |
| GO:1903409 | reactive oxygen species biosynthetic process | biological process | 4/71 | 56/14620 | 0.000 |
| GO:0101021 | estrogen 2-hydroxylase activity | molecular function | 2/71 | 3/15067 | 0.000 |
| GO:0101020 | estrogen 16-alpha-hydroxylase activity | molecular function | 3/71 | 3/15067 | 0.000 |
| GO:0072593 | reactive oxygen species metabolic process | biological process | 6/71 | 167/14620 | 0.000 |
| GO:0051707 | response to other organism | biological process | 9/71 | 533/14620 | 0.001 |
| GO:0051607 | defense response to virus | biological process | 5/71 | 143/14620 | 0.001 |
| GO:0050665 | hydrogen peroxide biosynthetic process | biological process | 2/71 | 10/14620 | 0.001 |
| GO:0044421 | extracellular region part | cellular component | 13/63 | 844/14551 | 0.000 |
| GO:0043207 | response to external biotic stimulus | biological process | 9/71 | 535/14620 | 0.001 |
| GO:0042572 | retinol metabolic process | biological process | 3/71 | 24/14620 | 0.000 |
| GO:0034308 | primary alcohol metabolic process | biological process | 3/71 | 37/14620 | 0.001 |
| GO:0034097 | response to cytokine | biological process | 9/71 | 541/14620 | 0.001 |
| GO:0033623 | regulation of integrin activation | biological process | 2/71 | 11/14620 | 0.001 |
| GO:0031349 | positive regulation of defense response | biological process | 6/71 | 223/14620 | 0.001 |
| GO:0019748 | secondary metabolic process | biological process | 3/71 | 32/14620 | 0.000 |
| GO:0016712 | oxidoreductase activity, acting on paired donors, with incorporation or reduction of molecular oxyge... | molecular function | 3/71 | 20/15067 | 0.000 |
| GO:0016101 | diterpenoid metabolic process | biological process | 3/71 | 40/14620 | 0.001 |
| GO:0009615 | response to virus | biological process | 6/71 | 192/14620 | 0.000 |
| GO:0009404 | toxin metabolic process | biological process | 3/71 | 12/14620 | 0.000 |
| GO:0008395 | steroid hydroxylase activity | molecular function | 3/71 | 11/15067 | 0.000 |
| GO:0008210 | estrogen metabolic process | biological process | 3/71 | 10/14620 | 0.000 |
| GO:0007159 | leukocyte cell-cell adhesion | biological process | 6/71 | 218/14620 | 0.001 |
| GO:0006955 | immune response | biological process | 12/71 | 856/14620 | 0.001 |
| GO:0006952 | defense response | biological process | 12/71 | 819/14620 | 0.001 |
| GO:0006721 | terpenoid metabolic process | biological process | 3/71 | 42/14620 | 0.001 |
| GO:0005615 | extracellular space | cellular component | 10/63 | 659/14551 | 0.000 |
| GO:0005576 | extracellular region | cellular component | 16/63 | 1237/14551 | 0.000 |
| GO:0002523 | leukocyte migration involved in inflammatory response | biological process | 2/71 | 8/14620 | 0.001 |
| GO:0002376 | immune system process | biological process | 17/71 | 1536/14620 | 0.001 |
| GO:0001523 | retinoid metabolic process | biological process | 3/71 | 39/14620 | 0.001 |

**Table S12.** Analysis of significant differences in miRNA expression between ISL group and CON group.

| MiRNA id^1^ | log2FoldChange^2^ | log2CPM^3^ | Pvalue |  |
| --- | --- | --- | --- | --- |
| Up | | | | |
| aae-miR-124 | 1.799 | -3.189 | 0.023 |  |
| aca-let-7c-5p | 1.162 | -2.150 | 0.021 |  |
| aca-miR-124a | 1.799 | -3.189 | 0.023 |  |
| aga-miR-124 | 1.799 | -3.189 | 0.023 |  |
| age-miR-124a | 1.905 | -2.992 | 0.019 |  |
| ame-miR-124 | 1.799 | -3.189 | 0.023 |  |
| api-miR-124 | 1.799 | -3.189 | 0.023 |  |
| asu-miR-124-3p | 1.799 | -3.189 | 0.023 |  |
| bbe-miR-124-3p | 1.799 | -3.189 | 0.023 |  |
| bfl-miR-124-3p | 1.799 | -3.189 | 0.023 |  |
| bma-miR-124 | 1.799 | -3.189 | 0.023 |  |
| bmo-miR-124 | 1.799 | -3.189 | 0.023 |  |
| bta-let-7c | 1.162 | -2.150 | 0.021 |  |
| bta-miR-124a | 1.799 | -3.189 | 0.023 |  |
| bta-miR-124b | 1.799 | -3.189 | 0.023 |  |
| bta-miR-1814c | 4.753 | -3.139 | 0.001 |  |
| bta-miR-187 | 2.522 | -3.975 | 0.044 |  |
| bta-miR-2284d | 1.631 | -2.609 | 0.007 |  |
| bta-miR-2310 | 4.753 | -3.139 | 0.001 |  |
| bta-miR-323 | 1.403 | -2.589 | 0.010 |  |
| bta-miR-654 | 4.927 | -3.156 | 0.012 |  |
| cbn-miR-124 | 1.799 | -3.189 | 0.023 |  |
| cbr-miR-124a | 1.799 | -3.189 | 0.023 |  |
| cel-miR-124-3p | 1.799 | -3.189 | 0.023 |  |
| cfa-let-7c | 1.162 | -2.150 | 0.021 |  |
| cfa-miR-124 | 1.799 | -3.189 | 0.023 |  |
| cfa-miR-187 | 2.522 | -3.975 | 0.044 |  |
| cfa-miR-323 | 1.403 | -2.589 | 0.010 |  |
| cgr-miR-124 | 1.799 | -3.189 | 0.023 |  |
| cgr-miR-187 | 2.522 | -3.975 | 0.044 |  |
| chi-let-7c-5p | 1.162 | -2.150 | 0.021 |  |
| chi-miR-124a | 1.799 | -3.189 | 0.023 |  |
| chi-miR-1814 | 4.844 | -3.072 | 0.001 |  |
| chi-miR-323a-3p | 1.403 | -2.589 | 0.010 |  |
| cin-miR-124-3p | 1.799 | -3.189 | 0.023 |  |
| cqu-miR-124 | 1.799 | -3.189 | 0.023 |  |
| crm-miR-124a | 1.799 | -3.189 | 0.023 |  |
| csa-miR-124 | 1.799 | -3.189 | 0.023 |  |
| cte-miR-124 | 1.799 | -3.189 | 0.023 |  |
| dan-miR-124 | 1.799 | -3.189 | 0.023 |  |
| der-miR-124 | 1.799 | -3.189 | 0.023 |  |
| dgr-miR-124 | 1.799 | -3.189 | 0.023 |  |
| dme-miR-124-3p | 1.799 | -3.189 | 0.023 |  |
| dmo-miR-124 | 1.799 | -3.189 | 0.023 |  |
| dpe-miR-124 | 1.799 | -3.189 | 0.023 |  |
| dps-miR-124 | 1.799 | -3.189 | 0.023 |  |
| dpu-miR-124 | 1.799 | -3.189 | 0.023 |  |
| dre-let-7c-5p | 1.162 | -2.150 | 0.021 |  |
| dre-miR-124-3p | 1.799 | -3.189 | 0.023 |  |
| dse-miR-124 | 1.799 | -3.189 | 0.023 |  |
| dsi-miR-124 | 1.799 | -3.189 | 0.023 |  |
| dvi-miR-124-3p | 1.799 | -3.189 | 0.023 |  |
| dwi-miR-124 | 1.799 | -3.189 | 0.023 |  |
| dya-miR-124 | 1.799 | -3.189 | 0.023 |  |
| eca-let-7c | 1.162 | -2.150 | 0.021 |  |
| eca-miR-124 | 1.799 | -3.189 | 0.023 |  |
| eca-miR-187 | 2.522 | -3.975 | 0.044 |  |
| eca-miR-323-3p | 1.403 | -2.589 | 0.010 |  |
| efu-let-7c | 1.162 | -2.150 | 0.021 |  |
| efu-miR-124 | 1.905 | -2.992 | 0.019 |  |
| efu-miR-323 | 1.403 | -2.589 | 0.010 |  |
| egr-miR-124a | 1.799 | -3.189 | 0.023 |  |
| emu-miR-124a | 1.799 | -3.189 | 0.023 |  |
| fru-miR-124 | 1.799 | -3.189 | 0.023 |  |
| gga-let-7c-5p | 1.162 | -2.150 | 0.021 |  |
| gga-miR-124a-3p | 1.905 | -2.992 | 0.019 |  |
| gga-miR-1692 | 2.170 | -2.490 | 0.003 |  |
| ggo-let-7c | 1.162 | -2.150 | 0.021 |  |
| ggo-miR-124a | 1.905 | -2.992 | 0.019 |  |
| ggo-miR-187 | 2.522 | -3.975 | 0.044 |  |
| ggo-miR-323a | 1.403 | -2.589 | 0.010 |  |
| hco-miR-124 | 1.799 | -3.189 | 0.023 |  |
| hhi-let-7c | 1.162 | -2.150 | 0.021 |  |
| hhi-miR-7641 | 1.750 | 1.099 | 0.000 |  |
| hme-miR-124 | 1.799 | -3.189 | 0.023 |  |
| hsa-let-7c-5p | 1.162 | -2.150 | 0.021 |  |
| hsa-miR-124-3p | 1.799 | -3.189 | 0.023 |  |
| hsa-miR-187-3p | 2.522 | -3.975 | 0.044 |  |
| hsa-miR-3168 | 2.468 | -2.627 | 0.003 |  |
| hsa-miR-323a-3p | 1.403 | -2.589 | 0.010 |  |
| hsa-miR-4426 | 2.330 | -3.801 | 0.024 |  |
| hsa-miR-4497 | 1.141 | -1.059 | 0.010 |  |
| hsa-miR-4508 | 2.877 | -3.806 | 0.015 |  |
| hsa-miR-4792 | 2.358 | 1.252 | 0.001 |  |
| hsa-miR-654-3p | 4.927 | -3.155 | 0.012 |  |
| hsa-miR-7641 | 1.990 | 0.641 | 0.000 |  |
| ipu-let-7c | 1.162 | -2.150 | 0.021 |  |
| ipu-miR-124a | 1.799 | -3.189 | 0.023 |  |
| isc-miR-124 | 1.799 | -3.189 | 0.023 |  |
| lgi-miR-124 | 1.799 | -3.189 | 0.023 |  |
| lla-miR-124a | 1.905 | -2.992 | 0.019 |  |
| lva-miR-124-3p | 1.799 | -3.189 | 0.023 |  |
| mdo-miR-124a-3p | 1.905 | -2.992 | 0.019 |  |
| Metazoa-novel-105-mature | 2.706 | -3.917 | 0.010 |  |
| Metazoa-novel-107-mature | 2.503 | -1.013 | 0.000 |  |
| Metazoa-novel-108-mature | 2.303 | -1.785 | 0.001 |  |
| Metazoa-novel-114-mature | 2.503 | -1.013 | 0.000 |  |
| Metazoa-novel-122-mature | 2.280 | -2.585 | 0.013 |  |
| Metazoa-novel-125-mature | 2.864 | -1.137 | 0.000 |  |
| Metazoa-novel-169-mature | 2.739 | -1.794 | 0.000 |  |
| Metazoa-novel-251-mature | 1.751 | -1.951 | 0.033 |  |
| Metazoa-novel-258-mature | 3.427 | -1.866 | 0.000 |  |
| Metazoa-novel-300-mature | 2.925 | 2.882 | 0.000 |  |
| Metazoa-novel-318-mature | 3.197 | 0.205 | 0.000 |  |
| Metazoa-novel-325-mature | 4.053 | -3.609 | 0.001 |  |
| Metazoa-novel-333-mature | 3.422 | -3.983 | 0.008 |  |
| Metazoa-novel-369-mature | 3.249 | -0.746 | 0.000 |  |
| Metazoa-novel-376-mature | 5.677 | -3.786 | 0.001 |  |
| Metazoa-novel-58-mature | 5.698 | -3.831 | 0.009 |  |
| mml-let-7c-5p | 1.162 | -2.150 | 0.021 |  |
| mml-miR-124a-3p | 1.905 | -2.992 | 0.019 |  |
| mml-miR-187-3p | 2.522 | -3.975 | 0.044 |  |
| mml-miR-323a-3p | 1.403 | -2.589 | 0.010 |  |
| mml-miR-654-3p | 4.927 | -3.156 | 0.012 |  |
| mmu-let-7c-5p | 1.162 | -2.150 | 0.021 |  |
| mmu-miR-124-3p | 1.799 | -3.189 | 0.023 |  |
| mmu-miR-187-3p | 2.522 | -3.975 | 0.044 |  |
| mmu-miR-323-3p | 1.403 | -2.589 | 0.010 |  |
| mmu-miR-6238 | 1.728 | -2.996 | 0.016 |  |
| mmu-miR-654-3p | 4.927 | -3.156 | 0.012 |  |
| mne-miR-187 | 2.522 | -3.975 | 0.044 |  |
| mse-miR-124 | 1.799 | -3.189 | 0.023 |  |
| nvi-miR-124 | 1.799 | -3.189 | 0.023 |  |
| oan-let-7c-5p | 1.162 | -2.150 | 0.021 |  |
| oan-miR-124a-1-3p | 1.799 | -3.189 | 0.023 |  |
| oar-let-7c | 1.162 | -2.150 | 0.021 |  |
| oar-miR-323a-3p | 1.403 | -2.589 | 0.010 |  |
| oar-miR-654-3p | 4.927 | -3.156 | 0.012 |  |
| oha-let-7c-5p | 1.162 | -2.150 | 0.021 |  |
| oha-miR-124 | 1.799 | -3.189 | 0.023 |  |
| oha-miR-124-3p | 1.799 | -3.189 | 0.023 |  |
| ola-let-7c | 1.162 | -2.150 | 0.021 |  |
| ola-miR-124-3p | 1.799 | -3.189 | 0.023 |  |
| pma-miR-124-3p | 1.799 | -3.189 | 0.023 |  |
| pmi-miR-124-3p | 1.799 | -3.189 | 0.023 |  |
| pol-let-7a-5p | 1.162 | -2.150 | 0.021 |  |
| pol-miR-124-3p | 1.799 | -3.189 | 0.023 |  |
| ppa-miR-124a | 1.905 | -2.992 | 0.019 |  |
| ppa-miR-187 | 2.522 | -3.975 | 0.044 |  |
| ppc-miR-124 | 1.799 | -3.189 | 0.023 |  |
| ppy-let-7c | 1.162 | -2.150 | 0.021 |  |
| ppy-miR-124 | 1.799 | -3.189 | 0.023 |  |
| ppy-miR-124a | 1.905 | -2.992 | 0.019 |  |
| ppy-miR-187 | 2.522 | -3.975 | 0.044 |  |
| ppy-miR-199b-5p | 1.170 | 0.703 | 0.026 |  |
| ppy-miR-323-3p | 1.403 | -2.589 | 0.010 |  |
| ppy-miR-654-3p | 4.927 | -3.156 | 0.012 |  |
| prd-miR-124-3p | 1.799 | -3.189 | 0.023 |  |
| ptr-let-7c | 1.162 | -2.150 | 0.021 |  |
| ptr-miR-124a | 1.905 | -2.992 | 0.019 |  |
| ptr-miR-323 | 1.403 | -2.589 | 0.010 |  |
| ptr-miR-654 | 4.927 | -3.156 | 0.012 |  |
| rno-let-7c-5p | 1.162 | -2.150 | 0.021 |  |
| rno-miR-124-3p | 1.799 | -3.189 | 0.023 |  |
| rno-miR-187-3p | 2.522 | -3.975 | 0.044 |  |
| rno-miR-323-3p | 1.403 | -2.589 | 0.010 |  |
| sko-miR-124-3p | 1.799 | -3.189 | 0.023 |  |
| sme-miR-124c-3p | 1.799 | -3.189 | 0.023 |  |
| spu-miR-124 | 1.799 | -3.189 | 0.023 |  |
| ssa-let-7c-5p | 1.162 | -2.150 | 0.021 |  |
| ssc-let-7c | 1.162 | -2.150 | 0.021 |  |
| ssc-miR-124a | 1.799 | -3.189 | 0.023 |  |
| ssc-miR-187 | 2.522 | -3.975 | 0.044 |  |
| ssc-miR-323 | 1.403 | -2.589 | 0.010 |  |
| str-miR-124-3p | 1.799 | -3.189 | 0.023 |  |
| tca-miR-124-3p | 1.799 | -3.189 | 0.023 |  |
| tgu-let-7c-5p | 1.162 | -2.150 | 0.021 |  |
| tgu-miR-124-3p | 1.799 | -3.189 | 0.023 |  |
| tni-miR-124 | 1.799 | -3.189 | 0.023 |  |
| tur-miR-124-3p | 1.799 | -3.189 | 0.023 |  |
| xbo-miR-124 | 1.799 | -3.189 | 0.023 |  |
| xtr-let-7c | 1.162 | -2.150 | 0.021 |  |
| xtr-miR-124 | 1.905 | -2.992 | 0.019 |  |
| **Down** | | | | |
| aae-miR-219 | -1.989 | -2.610 | 0.012 |  |
| aca-miR-219-5p | -1.989 | -2.610 | 0.012 |  |
| aca-miR-363-3p | -1.275 | -2.298 | 0.024 |  |
| aga-miR-219 | -1.989 | -2.610 | 0.012 |  |
| aja-miR-21 | -1.393 | 0.589 | 0.000 |  |
| ame-miR-219 | -1.989 | -2.610 | 0.012 |  |
| api-miR-219 | -1.989 | -2.610 | 0.012 |  |
| asu-miR-92-3p | -1.398 | -0.267 | 0.000 |  |
| bbe-miR-219-5p | -1.989 | -2.610 | 0.012 |  |
| bbe-miR-92b-3p | -2.074 | -2.899 | 0.011 |  |
| bfl-miR-219 | -1.989 | -2.610 | 0.012 |  |
| bfl-miR-92b-3p | -2.074 | -2.899 | 0.011 |  |
| bma-miR-92 | -1.398 | -0.267 | 0.000 |  |
| bta-miR-21-3p | -1.300 | 1.162 | 0.000 |  |
| bta-miR-219-5p | -1.989 | -2.610 | 0.012 |  |
| bta-miR-2285f | -1.020 | 3.842 | 0.000 |  |
| bta-miR-2285o | -2.276 | -1.813 | 0.000 |  |
| bta-miR-2285u | -1.911 | -1.476 | 0.001 |  |
| bta-miR-92b | -1.410 | 0.048 | 0.000 |  |
| ccr-miR-363 | -1.275 | -2.298 | 0.024 |  |
| ccr-miR-92b | -1.410 | 0.048 | 0.000 |  |
| cfa-miR-219-5p | -1.989 | -2.610 | 0.012 |  |
| cfa-miR-363 | -1.275 | -2.298 | 0.024 |  |
| cfa-miR-92b | -1.410 | 0.048 | 0.000 |  |
| cgr-miR-21-3p | -1.393 | 0.589 | 0.000 |  |
| cgr-miR-92b-3p | -1.410 | 0.048 | 0.000 |  |
| chi-miR-21-3p | -1.393 | 0.589 | 0.000 |  |
| chi-miR-219 | -1.989 | -2.610 | 0.012 |  |
| cin-miR-200-3p | -1.697 | -2.969 | 0.016 |  |
| cte-miR-219 | -1.989 | -2.610 | 0.012 |  |
| dan-miR-219 | -1.989 | -2.610 | 0.012 |  |
| der-miR-124 | -1.989 | -2.610 | 0.012 |  |
| der-miR-219 | -1.989 | -2.610 | 0.012 |  |
| dgr-miR-219 | -1.989 | -2.610 | 0.012 |  |
| dme-miR-219-5p | -1.989 | -2.610 | 0.012 |  |
| dmo-miR-219 | -1.989 | -2.610 | 0.012 |  |
| dpe-miR-219 | -1.989 | -2.610 | 0.012 |  |
| dps-miR-219 | -1.989 | -2.610 | 0.012 |  |
| dpu-miR-219 | -1.989 | -2.610 | 0.012 |  |
| dpu-miR-92 | -2.192 | -3.238 | 0.013 |  |
| dre-miR-219-5p | -1.989 | -2.610 | 0.012 |  |
| dre-miR-363-3p | -1.275 | -2.298 | 0.024 |  |
| dre-miR-429b | -1.697 | -2.969 | 0.016 |  |
| dre-miR-92b-3p | -1.410 | 0.048 | 0.000 |  |
| dse-miR-219 | -1.989 | -2.610 | 0.012 |  |
| dsi-miR-219 | -1.989 | -2.610 | 0.012 |  |
| dvi-miR-219-5p | -1.989 | -2.610 | 0.012 |  |
| dwi-miR-219 | -1.989 | -2.610 | 0.012 |  |
| dya-miR-219 | -1.989 | -2.610 | 0.012 |  |
| eca-miR-219-5p | -1.989 | -2.610 | 0.012 |  |
| eca-miR-363 | -1.275 | -2.298 | 0.024 |  |
| eca-miR-92b | -1.410 | 0.048 | 0.000 |  |
| efu-miR-21 | -1.393 | 0.589 | 0.000 |  |
| efu-miR-92b | -1.410 | 0.048 | 0.000 |  |
| fru-miR-219 | -1.989 | -2.610 | 0.012 |  |
| gga-miR-219a | -1.989 | -2.610 | 0.012 |  |
| ggo-miR-219 | -1.989 | -2.610 | 0.012 |  |
| ggo-miR-363 | -1.275 | -2.298 | 0.024 |  |
| hsa-miR-219a-5p | -1.989 | -2.610 | 0.012 |  |
| hsa-miR-363-3p | -1.275 | -2.298 | 0.024 |  |
| hsa-miR-92b-3p | -1.410 | 0.048 | 0.000 |  |
| ipu-miR-363 | -1.275 | -2.298 | 0.024 |  |
| ipu-miR-429b | -1.697 | -2.969 | 0.016 |  |
| ipu-miR-92b | -1.410 | 0.048 | 0.000 |  |
| isc-miR-219 | -1.989 | -2.610 | 0.012 |  |
| lva-miR-92b-3p | -1.355 | -0.297 | 0.000 |  |
| mdo-miR-219-5p | -1.989 | -2.610 | 0.012 |  |
| mdo-miR-92b-3p | -1.410 | 0.048 | 0.000 |  |
| Metazoa-novel-110-mature | -2.612 | -4.073 | 0.045 |  |
| Metazoa-novel-121-mature | -1.442 | -1.310 | 0.001 |  |
| Metazoa-novel-139-mature | -5.275 | -3.837 | 0.005 |  |
| Metazoa-novel-140-mature | -2.453 | -3.652 | 0.037 |  |
| Metazoa-novel-142-mature | -1.442 | -1.310 | 0.001 |  |
| Metazoa-novel-157-mature | -1.442 | -1.310 | 0.001 |  |
| Metazoa-novel-18-mature | -1.171 | -1.307 | 0.010 |  |
| Metazoa-novel-20-mature | -1.374 | -0.502 | 0.001 |  |
| Metazoa-novel-23-mature | -1.384 | -1.397 | 0.018 |  |
| Metazoa-novel-233-mature | -5.639 | -3.579 | 0.000 |  |
| Metazoa-novel-25-mature | -1.005 | -1.641 | 0.048 |  |
| Metazoa-novel-287-mature | -4.413 | -4.224 | 0.018 |  |
| Metazoa-novel-386-mature | -4.696 | -4.095 | 0.010 |  |
| Metazoa-novel-39-mature | -1.973 | -2.959 | 0.007 |  |
| Metazoa-novel-405-mature | -4.126 | -4.351 | 0.042 |  |
| Metazoa-novel-62-mature | -4.696 | -4.095 | 0.010 |  |
| Metazoa-novel-80-star | -1.072 | -1.939 | 0.022 |  |
| Metazoa-novel-87-mature | -1.171 | -1.307 | 0.010 |  |
| Metazoa-novel-90-mature | -1.065 | -1.845 | 0.028 |  |
| mml-miR-219 | -1.989 | -2.610 | 0.012 |  |
| mml-miR-219-5p | -1.989 | -2.610 | 0.012 |  |
| mml-miR-363-3p | -1.275 | -2.298 | 0.024 |  |
| mml-miR-92b-3p | -1.410 | 0.048 | 0.000 |  |
| mmu-miR-219a-5p | -1.989 | -2.610 | 0.012 |  |
| mmu-miR-21a-3p | -1.393 | 0.589 | 0.000 |  |
| mmu-miR-363-3p | -1.275 | -2.298 | 0.024 |  |
| mmu-miR-92b-3p | -1.410 | 0.048 | 0.000 |  |
| ngi-miR-219 | -1.989 | -2.610 | 0.012 |  |
| nlo-miR-219 | -1.989 | -2.610 | 0.012 |  |
| nvi-miR-219 | -1.989 | -2.610 | 0.012 |  |
| oan-miR-21-3p | -1.393 | 0.589 | 0.000 |  |
| oan-miR-219-5p | -1.989 | -2.610 | 0.012 |  |
| oan-miR-363-3p | -1.275 | -2.298 | 0.024 |  |
| odi-miR-92a | -1.472 | -0.485 | 0.000 |  |
| oha-miR-219-5p | -1.989 | -2.610 | 0.012 |  |
| oha-miR-363-3p | -1.275 | -2.298 | 0.024 |  |
| pma-miR-92a | -1.527 | -0.446 | 0.000 |  |
| pmi-miR-219-5p | -1.989 | -2.610 | 0.012 |  |
| pmi-miR-92b-3p | -1.355 | -0.297 | 0.000 |  |
| pmi-miR-92d-3p | -1.478 | -0.481 | 0.047 |  |
| pol-miR-219-5p | -1.989 | -2.610 | 0.012 |  |
| ppc-miR-235-3p | -1.398 | -0.267 | 0.000 |  |
| ppy-miR-219 | -1.989 | -2.610 | 0.012 |  |
| ppy-miR-219-5p | -1.989 | -2.610 | 0.012 |  |
| ppy-miR-363 | -1.275 | -2.298 | 0.024 |  |
| ppy-miR-92b | -1.410 | 0.048 | 0.000 |  |
| prd-miR-235-3p | -1.398 | -0.267 | 0.000 |  |
| ptr-miR-219-5p | -1.989 | -2.610 | 0.012 |  |
| ptr-miR-92b | -1.410 | 0.048 | 0.000 |  |
| rno-miR-21-3p | -1.393 | 0.589 | 0.000 |  |
| rno-miR-219a-5p | -1.989 | -2.610 | 0.012 |  |
| rno-miR-363-3p | -1.275 | -2.298 | 0.024 |  |
| rno-miR-92b-3p | -1.410 | 0.048 | 0.000 |  |
| sko-miR-219 | -1.989 | -2.610 | 0.012 |  |
| spu-miR-92c | -1.355 | -0.297 | 0.000 |  |
| ssa-miR-219a-5p | -1.989 | -2.610 | 0.012 |  |
| ssa-miR-219b-5p | -1.989 | -2.610 | 0.012 |  |
| ssa-miR-219c-5p | -1.989 | -2.610 | 0.012 |  |
| ssa-miR-21a-5p | -1.011 | 3.793 | 0.001 |  |
| ssa-miR-92b-3p | -1.410 | 0.048 | 0.000 |  |
| ssc-miR-363 | -1.275 | -2.298 | 0.024 |  |
| ssc-miR-92b-3p | -1.410 | 0.048 | 0.000 |  |
| tca-miR-219-5p | -1.989 | -2.610 | 0.012 |  |
| tch-miR-363-3p | -1.275 | -2.298 | 0.024 |  |
| tgu-miR-219a | -1.989 | -2.610 | 0.012 |  |
| tgu-miR-363-3p | -1.275 | -2.298 | 0.024 |  |
| tni-miR-219 | -1.989 | -2.610 | 0.012 |  |
| xbo-miR-219 | -1.895 | -2.545 | 0.011 |  |
| xbo-miR-92a | -1.418 | -0.271 | 0.000 |  |
| xtr-miR-219 | -1.989 | -2.610 | 0.012 |  |
| xtr-miR-363-3p | -1.275 | -2.298 | 0.024 |  |
| xtr-miR-92b | -1.410 | 0.048 | 0.000 |  |

^1^id：Mature miRNA id.

^2^logFoldChange：Differential multiple log2 conversion value.

^3^log2CPM：count-per-million log2 conversion value.

**Table S13.** Gene functions of 30 significantly enriched differential miRNAs targeting mRNA in the ISL group.

| GO.ID | Term | Ontology | GeneRatio^1^ | BgRatio^2^ | Pvalue |
| --- | --- | --- | --- | --- | --- |
| GO:0007155 | cell adhesion | biological process | 41/1304 | 237/27054 | 0.000 |
| GO:0007156 | homophilic cell adhesion via plasma membrane adhesion molecules | biological process | 24/1304 | 90/27054 | 0.000 |
| GO:0045944 | positive regulation of transcription from RNA polymerase II promoter | biological process | 78/1304 | 775/27054 | 0.000 |
| GO:0000122 | negative regulation of transcription from RNA polymerase II promoter | biological process | 57/1304 | 533/27054 | 0.000 |
| GO:0043547 | positive regulation of GTPase activity | biological process | 30/1304 | 197/27054 | 0.000 |
| GO:0007018 | microtubule-based movement | biological process | 16/1304 | 70/27054 | 0.000 |
| GO:0042472 | inner ear morphogenesis | biological process | 12/1304 | 44/27054 | 0.000 |
| GO:0006355 | regulation of transcription, DNA-templated | biological process | 86/1304 | 1051/27054 | 0.000 |
| GO:0007507 | heart development | biological process | 22/1304 | 155/27054 | 0.000 |
| GO:0035023 | regulation of Rho protein signal transduction | biological process | 15/1304 | 80/27054 | 0.000 |
| GO:0005634 | nucleus | cellular component | 270/1304 | 3186/27054 | 0.000 |
| GO:0005654 | nucleoplasm | cellular component | 177/1304 | 2246//27054 | 0.000 |
| GO:0005737 | cytoplasm | cellular component | 194/1304 | 2612/27054 | 0.000 |
| GO:0005829 | cytosol | cellular component | 167/1304 | 2176/27054 | 0.000 |
| GO:0016604 | nuclear body | cellular component | 35/1304 | 262/27054 | 0.000 |
| GO:0005623 | cell | cellular component | 59/1304 | 598/27054 | 0.000 |
| GO:0005886 | plasma membrane | cellular component | 171/1304 | 2418/27054 | 0.000 |
| GO:0098978 | glutamatergic synapse | cellular component | 26/1304 | 213/27054 | 0.000 |
| GO:0030027 | lamellipodium | cellular component | 16/1304 | 106/27054 | 0.000 |
| GO:0005911 | cell-cell junction | cellular component | 19/1304 | 142/27054 | 0.000 |
| GO:0005515 | protein binding | molecular function | 269/1304 | 2880/27054 | 0.000 |
| GO:0005509 | calcium ion binding | molecular function | 73/1304 | 576/27054 | 0.000 |
| GO:0003677 | DNA binding | molecular function | 109/1304 | 1122/27054 | 0.000 |
| GO:0005524 | ATP binding | molecular function | 120/1304 | 1328/27054 | 0.000 |
| GO:0005085 | guanyl-nucleotide exchange factor activity | molecular function | 24/1304 | 141/27054 | 0.000 |
| GO:0003777 | microtubule motor activity | molecular function | 16/1304 | 70/27054 | 0.000 |
| GO:0008017 | microtubule binding | molecular function | 26/1304 | 171/27054 | 0.000 |
| GO:0000978 | RNA polymerase II core promoter proximal region sequence-specific DNA binding | molecular function | 40/1304 | 380/27054 | 0.000 |
| GO:0003700 | transcription factor activity, sequence-specific DNA binding | molecular function | 53/1304 | 573/27054 | 0.000 |
| GO:0043565 | sequence-specific DNA binding | molecular function | 44/1304 | 444/27054 | 0.000 |

^1^ GeneRatio: The number of target genes in this GO entry/the number of genes with GO annotations in the target gene.

^2^ BgRatio: The number of genes in the GO entry of this entry/the number of genes with GO annotations in all genes.

**
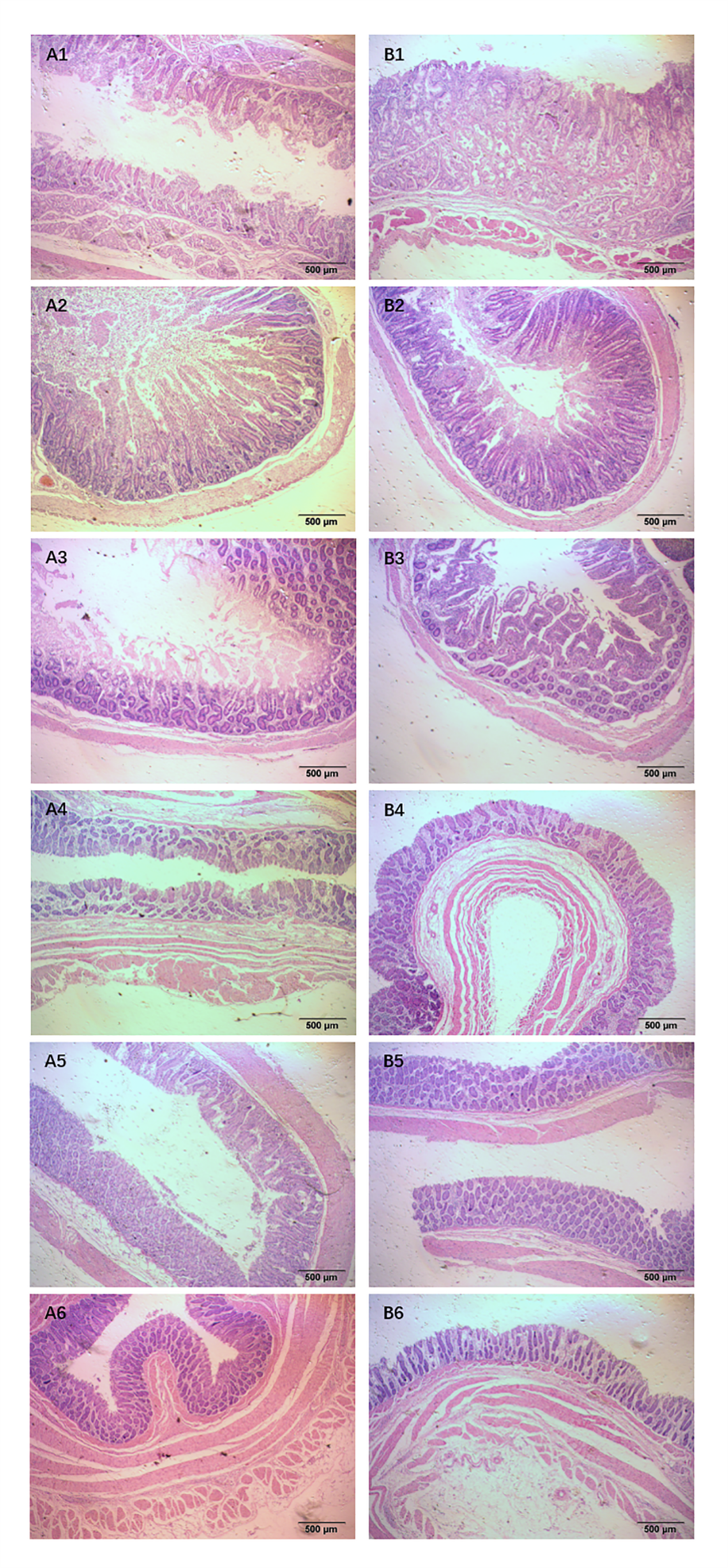
Figure S1.** Histomorphology of intestinal segments in fattening sheep fed with ISL diet **(A),** CON **(B),** ISL **(1)**, Duodenum **(2),** Jejunum **(3)**, Ileum **(4),** Cecum **(5)**, Colon **(6),** Rectum.
